# Abnormal lipid processing but normal long-term repopulation potential of *myc−/−* hepatocytes

**DOI:** 10.18632/oncotarget.8856

**Published:** 2016-04-20

**Authors:** Lia R. Edmunds, P. Anthony Otero, Lokendra Sharma, Sonia D'souza, James M. Dolezal, Sherin David, Jie Lu, Lauren Lamm, Mahesh Basantani, Pili Zhang, Ian J. Sipula, Lucy Li, Xuemei Zeng, Ying Ding, Fei Ding, Megan E. Beck, Jerry Vockley, Satdarshan P. S. Monga, Erin E. Kershaw, Robert M. O'Doherty, Lisa E. Kratz, Nathan A. Yates, Eric P. Goetzman, Donald Scott, Andrew W. Duncan, Edward V. Prochownik

**Affiliations:** ^1^ Division of Hematology/Oncology, Children's Hospital of Pittsburgh of the University of Pittsburgh Medical Center, Pittsburgh, PA, USA; ^2^ Department of Molecular Genetics and Developmental Biology, University of Pittsburgh, Pittsburgh, PA, USA; ^3^ Department of Pathology, McGowan Institute for Regenerative Medicine, University of Pittsburgh, Pittsburgh, PA, USA; ^4^ Division of Endocrinology and Metabolism, Department of Medicine, University of Pittsburgh Medical Center, Pittsburgh, PA, USA; ^5^ Division of Endocrinology, Diabetes and Bone Disease, Department of Medicine, Mt. Sinai School of Medicine, New York, NY, USA; ^6^ Biomedical Mass Spectrometry Center, University of Pittsburgh Schools of the Health Sciences, Pittsburgh, PA, USA; ^7^ Department of Biostatistics, Graduate School of Public Health, University of Pittsburgh, Pittsburgh, PA, USA; ^8^ Division of Medical Genetics, Children's Hospital of UPMC, The University of Pittsburgh Medical Center, Pittsburgh, PA, USA; ^9^ Department of Pathology, Division of Experimental Pathology, University of Pittsburgh, Pittsburgh, PA, USA; ^10^ Laboratory of Biochemical Genetics Kennedy Krieger Institute, Johns Hopkins University School of Medicine, Baltimore, MD, USA; ^11^ Department of Cell Biology, University of Pittsburgh School of Medicine, Pittsburgh, PA, USA; ^12^ The University of Pittsburgh Cancer Institute, Pittsburgh, PA, USA; ^13^ Biotechnology Program, Center for Biological Sciences, Central University of Bihar, Bihar, India

**Keywords:** hereditary tyrosinemia, cytochrome p450, electron transport chain, NAFLD, NASH

## Abstract

Establishing c-Myc's (Myc) role in liver regeneration has proven difficult particularly since the traditional model of partial hepatectomy may provoke an insufficiently demanding proliferative stress. We used a model of hereditary tyrosinemia whereby the affected parenchyma can be gradually replaced by transplanted hepatocytes, which replicate 50-100-fold, over several months. Prior to transplantation, livers from *myc−/−* (KO) mice were smaller in young animals and larger in older animals relative to *myc+/+* (WT) counterparts. KO mice also consumed more oxygen, produced more CO_2_ and generated more heat. Although WT and KO hepatocytes showed few mitochondrial structural differences, the latter demonstrated defective electron transport chain function. RNAseq revealed differences in transcripts encoding ribosomal subunits, cytochrome p450 members and enzymes for triglyceride and sterol biosynthesis. KO hepatocytes also accumulated neutral lipids. WT and KO hepatocytes repopulated recipient tyrosinemic livers equally well although the latter were associated with a pro-inflammatory hepatic environment that correlated with worsening lipid accumulation, its extracellular deposition and parenchymal oxidative damage. Our results show Myc to be dispensable for sustained *in vivo* hepatocyte proliferation but necessary for maintaining normal lipid homeostasis. *myc−/−* livers resemble those encountered in non-alcoholic fatty liver disease and, under sustained proliferative stress, gradually acquire the features of non-alcoholic steatohepatitis.

## INTRODUCTION

A direct role for the c-Myc (Myc) oncoprotein in promoting and/or sustaining neoplastic growth was first demonstrated over 35 years ago [1]. This has been subsequently supported by studies documenting recurrent *MYCC* de-regulation in human tumors, from animal models of Myc over-expression and from demonstrations that Myc silencing in these models promotes tumor regression [2, 3]. Although Myc supervises many of the pathways that are commonly perturbed in cancer such as those regulating differentiation, proliferation, survival and metabolism [4, 5], its precise role in these processes and its function(s) in normal cells have been somewhat more controversial and, at times, subject to conflicting experimental outcomes.

A requirement for Myc is well-established in the developing mouse where *myc−/−* embryonic lethality occurs at E9.5-10.5 as a result of placental insufficiency and cardiac and neural tube defects [6]. Postnatally, Myc silencing is compatible with long-term survival and is associated with only mild and reversible toxicities in proliferative tissues such as the bone marrow and gastrointestinal track [7]. Moreover, haplo-insufficiency of Myc actually slows the onset of numerous age-related pathologies and prolongs life span [8]. In contrast, the *in vitro* proliferation of primary murine embryonic fibroblasts declines progressively as Myc levels are reduced [9]. Thus, the consequences of Myc loss in normal cells appear to be more tissue- and context-dependent and variable than in transformed cells [9].

Myc's presumptive role in hepatic regeneration rests partially on the observation that it is induced within minutes of partial hepatectomy (PH) and silenced following the restoration of normal liver mass over the ensuing 7-8 days [10]. However, the actual requirement for Myc in this process has varied among different studies, which have not always assessed identical parameters or used similar methods for achieving *myc* gene deletion [11–13]. For example, while Sanders *et al.* used albumin-driven expression of Cre recombinase (Alb-Cre) to excise *myc* from hepatocytes, Baena *et al.* used interferon-mediated induction of *mx*-Cre, resulting in extensive *myc* deletion in cells other than hepatocytes, including other non-hepatocyte liver cells, and rendering interpretation of the ensuing hepatic phenotype equivocal [11, 12]. These reports also relied exclusively on PH, which, in addition to the short duration of the regenerative response, is further hampered by the fact that fewer than 2 population doublings occur during the process [11]. Additionally, previous reports relying on PH were also largely dependent on indirect measurements of liver regeneration such as the expression of Ki-67, PCNA and cyclin A levels or liver:body weight ratios [11, 12, 14]. For these reasons, we sought here to assess Myc's role in hepatocyte proliferation by utilizing a novel mouse model of liver regeneration that imposes fewer proliferative or temporal restrictions.

Type I hereditary tyrosinemia is a metabolic disorder caused by defective production of fumarylacetoacetate hydrolase (FAH), which catalyzes the final step in hepatic tyrosine catabolism. In HT, the toxic upstream metabolites fumaryl- and maleyl-acetoacetate accumulate and cause liver failure [15]. Deletion of the murine *fah* gene faithfully mimics the human disease [16]. Hepatocyte death in *fah−/−* animals can be prevented with the drug 2- (2-nitro-4-trifluoro-methyl-benzoyl)-1,3-cyclo-hexanedione (NTBC), which inhibits p-hydroxyphenylpyruvate dioxygenase, a more proximal enzyme that catalyzes the second step in the tyrosine catabolic pathway [16]. Alternatively, *fah−/−* animals can be rescued by the intrasplenic injection of *fah+/+* hepatocytes, which migrate to the recipient liver and expand 50-100-fold over several weeks-months as they replace the defective parenchyma [17, 18].

Here, we studied livers or purified hepatocytes from mice in which “floxed” *myc* alleles were excised in the perinatal period in response to Alb-Cre expression (hereafter referred to as KO mice) or from littermate controls (WT mice). We report heretofore unappreciated phenotypes and gene expression differences in KO hepatocytes both prior to and following transplant into *fah−/−* recipients. We also show WT and KO hepatocytes to be equally proficient at long-term repopulation of *fah−/−* livers, either when transplanted individually or competitively. The ability of WT and KO hepatocytes to proliferate identically under highly demanding conditions may explain why a more global long-term silencing of Myc is associated with only mild side effects or may even be beneficial [8]. Oncogene “addiction”, a frequent feature of Myc-over-expressing cancers [19], may thus impose unique cellular changes that are necessary for maintaining transformed but not normal states of proliferation.

## RESULTS

### Characterization of livers and hepatocytes from WT and KO mice

We first verified excision of *myc^fl/fl^* alleles from purified KO hepatocytes (Figure S1A-S1E). RNAseq from the same cell populations further confirmed a > 90% reduction in the number of reads across exons 2 and 3 relative to those from WT hepatocytes (Figure S1F).

Although body weights of WT and KO mice were indistinguishable (not shown), liver:total body weight ratios differed, being lower in young (< 1 month) KO animals and higher in older (> 3 months) animals (Figure S2A). This was not associated with any differences in liver histology as assessed by hematoxylin and eosin (H&E) staining (Figure S2B). WT and KO hepatocyte sizes were also similar (Figure S2C) indicating that, as previously reported, KO livers contain fewer rather than smaller hepatocytes to account for their reduced mass [8, 9]. We discuss below possible reasons for the larger liver:body mass ratio of older KO mice.

*myc−/−* rat fibroblasts have dysfunctional mitochondria and a profound energy deficit [20, 21]. To rectify this, they up-regulate pyruvate dehydrogenase (PDH), presumably to maximize pyruvate conversion to acetyl CoA [20]. They also constitutively up-regulate AMP-activated protein kinase (AMPK), which responds to reduced ATP:AMP ratios by down-regulating energy-consuming processes and up-regulating energy-generating ones as fatty acid β-oxidation (FAO) [20]. WT and KO livers showed no significant differences in any of these properties except for variable but increased rates of FAO in the latter (Figure S2D-S2H).

Finally, we noted that while Myc alters the susceptibility to multiple intrinsic and environmental pro-apoptotic factors [22, 23], no increase in apoptosis in KO livers was found based on lack of cleaved caspase 3 (Figure S2I).

### Differences in metabolism and mitochondrial function of KO mice

**Figure 1:**
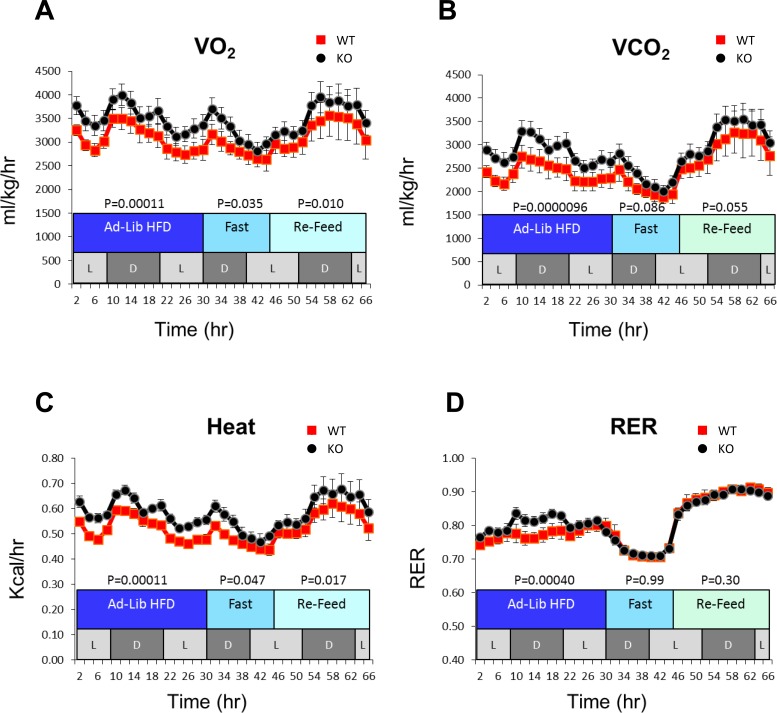
Increased metabolic activity of KO mice Mice were maintained in metabolic cages that quantified oxygen consumption rate (VO_2_), carbon dioxide production rate (VCO_2_) and heat production (6-8 mice per group). Mice of similar weights were initially maintained on an *ad lib* high-fat diet followed by a period of fasting and subsequent re-feeding with standard chow plus 5% glucose-containing water over the course of the experiment. L and D indicate cyclic periods of light and dark. Other monitoring showed no differences in the overall activity, food consumption, total fat mass or glucose tolerance between groups of WT and KO mice (not shown).

KO mice were noted to have increased oxygen consumption, carbon dioxide production and heat generation (Figure 1) when maintained on a high fat diet. These studies suggested that the increased metabolism of KO mice is due to abnormalities in lipid metabolism.

In addition to altering cellular metabolism, Myc also affects mitochondrial structure and function [20, 21, 24]. Although blue native gel electrophoresis (BNGE) showed no significant differences in electron transport chain (ETC) complexes I-IV and Complex V from WT and KO livers (Figure S3), higher ATPase activity of Complex V monomers and dimers (V_m_ and V_d_) was noted in the latter (Figure S3H and S3I).

**Figure 2:**
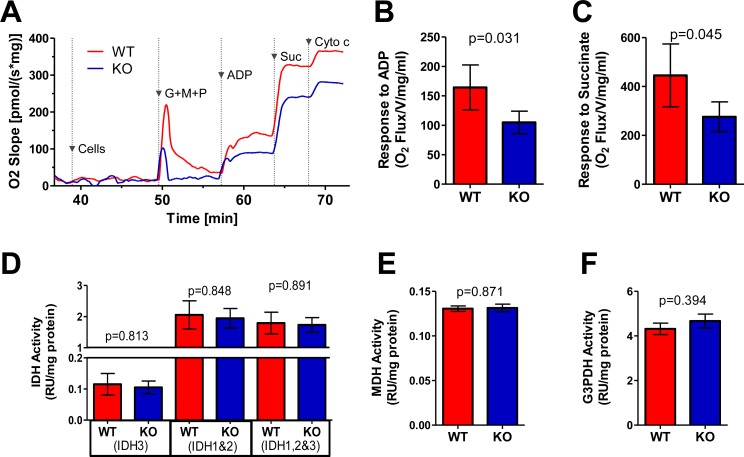
ETC function of WT and KO livers **A.** Typical respirometry profiles from WT and KO liver homogenates. Arrowheads indicate the time of addition of glutamate, malate and pyruvate (G,M,P), ADP and succinate (Suc). WT and KO liver preparations were simultaneously assayed in parallel with 10 sets of mice. Cytochrome c was added to test mitochondrial outer membrane integrity. Note that the large, spike in O_2_ flux seen upon adding G,M,P is an artifact resulting from injecting a large volume of diluted substrates and transiently disturbing the O_2_ concentration. **B.** Graphic representation of the ADP responses in mitochondria prepared from 7 sets of WT and KO livers assayed as shown in **A. C.** Graphic representation of succinate responses. The results in **A.**-**C.** were adjusted to account for minor differences in total protein concentrations. **D.**-**F.** Assays of selective TCA cycle dehydrogenases [25], including: NAD+- and NADP+-dependent IDHs (IDH3 and IDH1+2) **D.**; MDH **E.** and G3PDH **F.** See Figure S3E for similar assays performed for SDH (Complex II). Assays were performed on 8 sets of WT and KO livers from littermate controls.

Respirometry measurements showed basal O_2_ flux of WT and KO liver mitochondria to be low but otherwise equivalent prior to and after priming with the Complex I substrates glutamate, pyruvate and malate (G,M,P) (Figure 2A). However, the magnitude of O_2_ flux change in response to ADP was less pronounced in KO mitochondria (Figure 2A and 2B), indicating that they reduced molecular oxygen to water *via* complex IV less efficiently. The provision of succinate resulted in additional but similarly unequal increases in O_2_ flux. This indicated that the ability of Complex II (succinate dehydrogenase [SDH]) to drive additional electron flow was unable to compensate for the Complex I defect of KO mitochondria (Figure 2A and 2C).

We also quantified the activities of several mitochondrial dehydrogenases other than Complex II (Figure S3E), which donate electrons to the ETC [25]. WT and KO liver-derived mitochondria had equal levels of isocitrate dehydrogenases (IDH) 1-3, malate dehydrogenase (MDH) and glycerol 3-phosphate dehydrogenase (G3PDH) (Figure 2D-2F). Taken together we conclude that, while KO liver mitochondria have defects in Oxphos, they are not severe enough to compromise ATP production.

We next employed a recently described targeted proteomics assay using liquid chromatography-tandem mass spectrometry to assess the relative abundance of the 93 known subunits of the ETC plus an additional 46 mitochondrial metabolism-related proteins [25]. Based on a p-value cut-off of 0.05, none of the quantified proteins exhibited statistically significant differences between the two groups (Table S1).

Next, an unbiased mass spectrometry-based approach was used to compare the relative abundance of a subset of proteins comprising ~30% of the mitochondrial proteome [25]. In total, 2439 peptide ions (features) were matched to 377 mitochondrial proteins. Using a Student's *t*-test with a cut off of *p* < 0.05, we again found there to be no significant differences between WT and KO livers with respect to the relative amounts of any of these proteins (Table S2). Together with the results shown in Figure S3, we conclude there to be no major quantitative differences in the composition of the mitochondrial proteomes of WT and KO livers.

### RNAseq analysis of WT and KO hepatocytes

**Figure 3:**
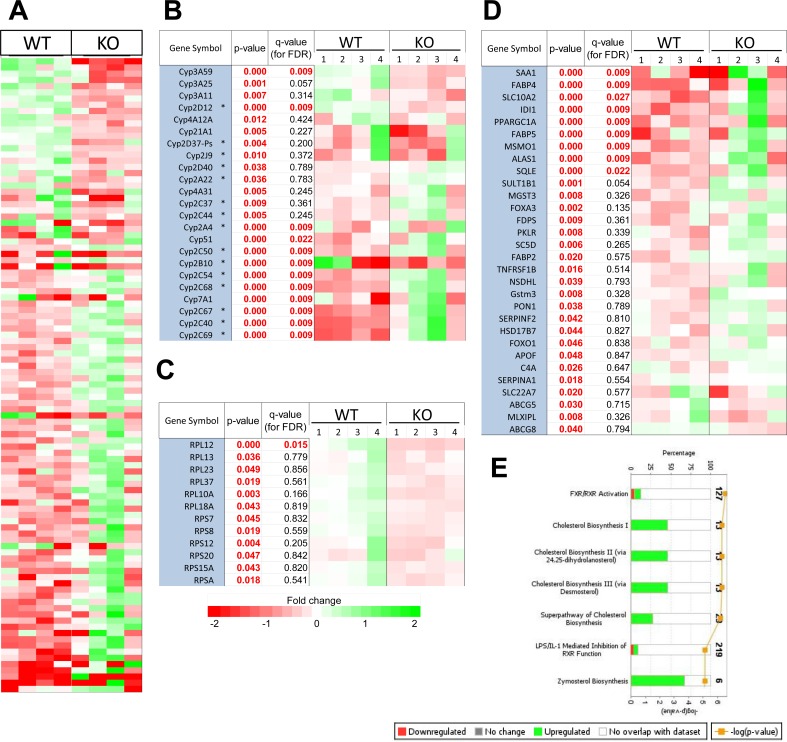
Transcript differences between WT and KO hepatocytes **A.** RNAseq results from 4 individual sets each of WT and KO hepatocytes. The 102 differentially expressed transcripts with adjusted FDR *q* < 0.05 are shown. **B.** Differences in cyp450 member transcript expression between WT and KO hepatocytes. Those with *q* < 0.05 were taken from A whereas the others represent significant differences at the level of *p* < 0.05 among other members of the cyp450 family. Those encoding members of the CLAN 2 family [26] are indicated by asterisks. **C.** Differentially expressed transcripts encoding ribosomal proteins (*p* < 0.05) are listed. RPL12 is from A. **D.** Differential gene expression profiling of genes involved in the top 7 deregulated pathways identified by Ingenuity Pathway Analysis. Note that the cyp450 members shown in B are not repeated here but were included and found to be significant in the pathway analysis of cholesterol metabolism. **E.** The top 7 classifications of all significantly deregulated (*p* < 0.05) transcripts according to their involvement in specific pathways based on Ingenuity profiling. The top axis represents the percentage of genes comprising that pathway whose transcripts were differentially expressed between WT and KO hepatocytes. Green bars indicate transcripts that were up-regulated in KO hepatocytes and red bars indicate down-regulated transcripts. Some transcripts are included in more than one pathway. Orange circles indicate the log_10_ p value indicating the probability that the differentially expressed transcripts within a specific pathway would have been selected randomly. Numbers on the right indicate the number of transcripts assigned to the indicated pathway.

We performed RNAseq on isolated hepatocytes from WT and KO animals of comparable ages (ca. 17-18 weeks). After adjusting for false discovery rate (FDR), we identified 102 transcript differences, with 35 being up-regulated in KO hepatocytes and 67 being down-regulated (*q* values < 0.05, Figure 3A). The largest subset was comprised of 12 of the ~103 member cytochrome p450 (cyp450) family (Figure 3B) [26]. Re-evaluation of our data identified 11 additional members that were initially excluded for not meeting false discovery rate (FDR) criteria but which still demonstrated significant differences between the two groups (*p* < 0.05) (Figure 3B). 15 of the 23 differentially expressed cyp450 transcripts (65%) were encoded by the CLAN2 family whose 37 members are particularly relevant for the regulation of sterol, bile acid and eicosinoid metabolism [26].

In keeping with Myc's role in regulating ribosomal biogenesis [27], 12 of the ~80 known transcripts (~15%) encoding ribosomal proteins were down-regulated in KO hepatocytes (Figure 3C) although only one of these (RPL12) was significantly regulated at the *q* < 0.05 level. Thus, endogenous Myc maintains normal levels of a subset of ribosomal protein transcripts.

Ingenuity Pathway Analysis of all differentially expressed transcripts of significance (*p* < 0.05) identified specific functional classes of genes that were not necessarily fully represented in Figure 3A. Of 642 pathways queried, 5 of the top 7 involved the regulation of cholesterol, sterol or bile acid synthesis (Figure 3D and 3E). The remaining 2 related to farnesoid X receptor (FXR)- and the liver X receptor (LXR) or their regulation by lipopolysaccharide and/or interleukin 1, which are also activated by oxysterols and bile acids [28]. Thus, KO hepatocytes also up-regulate multiple pathways involved directly in sterol and bile acid bio-synthesis.

WT and KO hepatocytes did not differentially express genes potentially capable of complementing the loss of Myc such as N-Myc and L-Myc and non-Myc family-related genes such as Myct1, HMG-IY and serine hydroxymethyltransferase [29, 30] (Figure S4A). Transcripts encoding the “Mlx family” of bHLH-ZIP transcription factors were also unchanged save for a modest (~30%) decrease in those encoding Mlxip1/MondoB/ChREBP in KO cells [31], which was not manifested at the protein level (Figure S4B).

### Abnormal regulation of triglycerides and sterols in KO livers

Myc inhibition *in vitro* increases FAO and neutral lipid storage [20, 24], which is consistent with the increased FAO rate in KO livers (Figure S2H). Our RNAseq results and the fact that lipid droplets also contain cholesterol suggested that sterol and/or bile acid metabolism might be compromised in KO hepatocytes particularly since bile acids derive from sterols and can regulate sterol and triglyceride biosynthetic pathways [32]. Indeed, livers of fasted KO mice contained higher triglyceride levels as well as more numerous and larger neutral lipid droplets (Figure S5A and S5B and S6A and S6B). A trend toward lower fasting cholesterol levels was also noted and increased significantly following re-feeding (Figure S5C). KO hepatocytes also contained significantly higher levels of transcripts for squalene epoxidase (SQLE), sterol C-4 methyloxidase (SC4MOL), 3β-hydroxysteroid dehydrogenase (NSDHL) and sterol C-5 desaturase (SC5DL) [1.7-fold, 2.0-fold, 1.3-fold and 1.4-fold, respectively (Figure S5D)]. However, profiling a series of intermediates in the biosynthetic pathway leading from lanosterol to cholesterol, and including the bile acid intermediate cholestenol, showed no differences between WT and KO mice when adjusted either to total protein or total sterol content (Figure S5E and S5F).

Bile acids consist of conjugates between taurine or glycine and cholic or chenodeoxycholic acid [33]. When measured in the livers of non-fasted animals, secondary bile acids, which are formed within the small intestine by the action of enteric bacteria, make a larger contribution to the total bile acid pool. Therefore, we measured total bile acid levels in the livers of fasted WT and KO mice. As seen in Figure S5G, no significant differences were observed.

### WT and KO hepatocytes have equivalent repopulation capacity

Mice with tyrosinemia can be rescued by the intrasplenic injection of FAH*+* donor hepatocytes, particularly when performed in immune-compromised *fah−/−* FRG-NOD recipients [34]. This approach therefore provides a superior alternative to PH as the transplanted hepatocytes are subject to a more intense and prolonged replicative stress.

**Figure 4:**
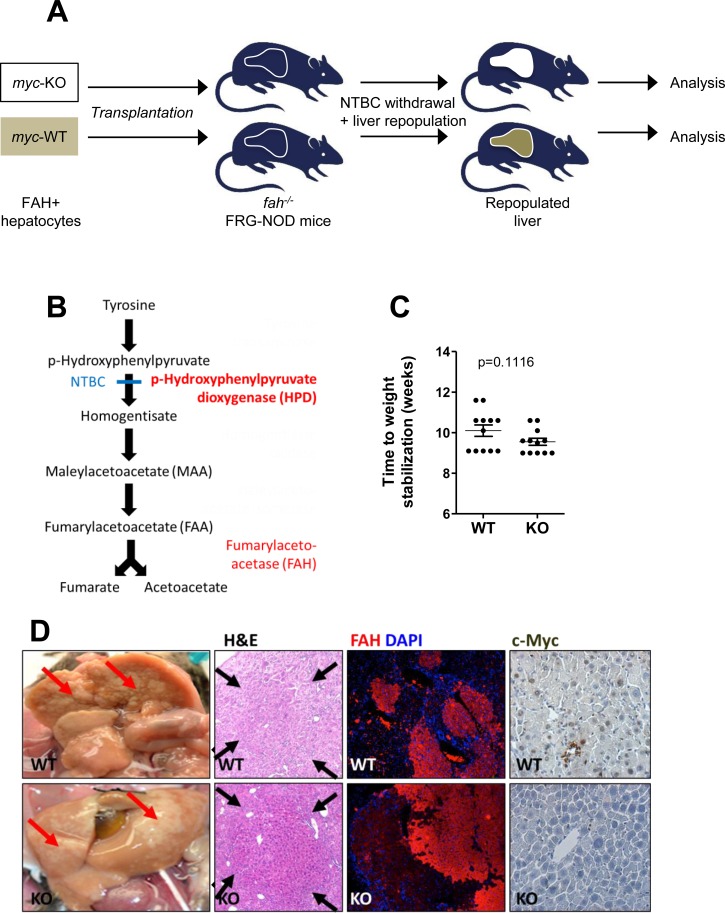
Rescue of FGR-NOD mice with *fah+/+* WT and KO hepatocytes occurs at equivalent rates **A.** Schematic for repopulating *fah−/−* NOD-SCID mice with WT or KO donor *fah+/+* hepatocytes **B.** Pathway of hepatic tyrosine catabolism. FAH's absence causes accumulation of the toxic upstream intermediates maleyl- and fumarylacetoacetate. Animals can be rescued by blocking the more proximal enzyme hydroxyphenylpyruvate dioxygenase with NTBC or by transplanting *fah+/+* hepatocytes. **C.** 3×10^5^ hepatocytes from WT or KO mice (both *fah+/+*) were inoculated intrasplenically into recipient *fah−/−* FRG-NOD mice. 4 days later, NTBC cycling began until body weight stabilized at > 100% of the pre-transplant weight. No differences were seen in the times needed to achieve NTBC independence and body weight stabilization according to a Students' *t*-test. **D.** Gross appearance of recipient FRG-NOD livers 12-14 weeks after transplantation of WT or KO hepatocytes. Livers have been flushed with PBS to facilitate visualization of regenerating nodules (arrows). H&E staining of post-transplant livers showing regenerating nodules of donor cells (arrows). FAH immuno-staining of donor-derived *fah+/+* nodules (red) in recipient *fah−/−* FRG-NOD mice. Note the lack of staining of adjacent, *fah−/−* recipient hepatocytes. Tissues were counterstained with DAPI (blue). Myc immuno-staining of regenerating hepatic nodules. Numerous representative Myc-positive nuclei in a regenerating nodule following transplantation with WT hepatocytes are shown in the top panel. Note the absence of Myc in a similar nodule repopulated with KO hepatocytes (lower panel). At least 10 sets of repopulated livers were examined for these studies.

We injected FRG-NOD mice with 3×10^5^ WT or KO hepatocytes and withdrew NTBC 4d post-transplant to facilitate recipient liver repopulation (Figure 4A and 4B). Because NTBC discontinuation is associated with weight loss, it was re-initiated whenever body weights dropped by > 20% and discontinued permanently when weights stabilized at > 100% of pre-transplant values. The time needed to permanently re-establish pre-transplant weights was similar in the two groups suggesting that WT and KO hepatocytes possessed equal repopulation potential (Figure 4C). This was supported by the presence of numerous macroscopic and microscopic foci of regenerating hepatocytes in both groups of animals (Figure 4D) and by extensive focal FAH immuno-positivity (Figure 4D, FAH). Additional immuno-histochemical staining showed Myc-positive hepatocytes only in regenerating nodules derived from WT donors (Figure 4D, c-Myc). Thus, liver regeneration by KO hepatocytes was not due to the selective expansion of a minority sub-population of hepatocytes with non-excised *myc* loci.

**Figure 5:**
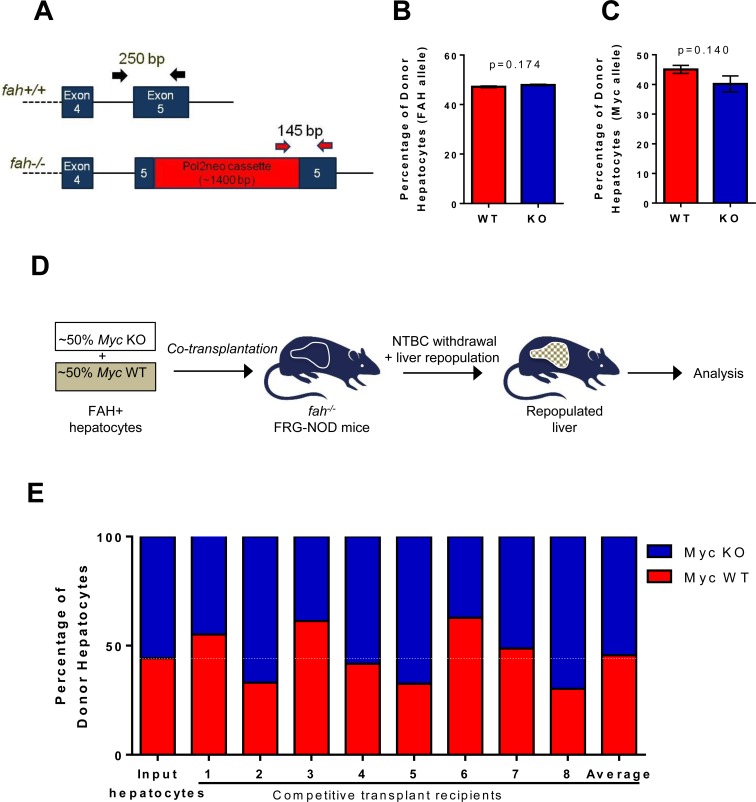
WT and KO hepatocytes are equally proficient at re-populating the hepatic parenchyma **A.** PCR-based strategy to distinguish *fah+/+* donor and *fah−/−* recipient alleles. **B.** Hepatocytes were isolated from 11 recipient animals 17-18 weeks after transplanting with WT or KO hepatocytes and ~6 weeks after NTBC discontinuation. PCR of *fah* alleles showed no difference by Students' *t*-test in the contribution of WT and KO *myc* alleles to the repopulated liver. **C.** The strategy depicted in Figure S1B and C (black and blue arrows) was used to amplify the *myc* alleles from the same hepatocyte samples used in B. **D.** Schematic for competitive transplants of Myc WT and KO hepatocytes. **E.** 8 FRG-NOD mice were transplanted with a ~1:1 mixture of 1.5×10^5^ hepatocytes each from WT and KO donors (input) and allowed to achieve NTBC independence. Following repopulation, PCR quantification of *fah* alleles again showed that the donor populations comprised ~40% of the total hepatocyte mass (not shown). This was confirmed by amplification of the three *myc* alleles. Shown here is the proportion of the *donor* population in each animal that was comprised of Myc WT and KO donor hepatocytes. Note that the average allelic composition of this group was identical to that of the input population. Each set of PCR reactions was performed in triplicate for each population of hepatocytes.

Quantification of *fah^+/+^* and *fah^−/−^* alleles in hepatocytes isolated from repopulated livers established that *fah^+/+^* donor cells comprised ~40-50% of the entire hepatocyte population (Figure 5A and 5B). This was independently confirmed using the PCR-based strategy shown in Figure S1B and S1C (black and blue arrows) to amplify different sized segments of donor and recipient *myc* loci.

A more sensitive quantification of proliferative potential was obtained with a competitive re-population assay performed ~18 weeks after transplanting a mixed population of WT and KO donor hepatocytes (Figure 5D). PCR of *fah* alleles (Figure 5A) again indicated transplant efficiency to be ~40-50% (not shown). PCR for *myc* alleles (Figure S1A-S1C) showed that, on average, their individual contributions precisely reflected those of the input populations (Figure 5E). KO hepatocytes therefore are not at a proliferative disadvantage even when competing directly with their WT counterparts.

### Abnormal neutral lipid storage following transplantation with KO hepatocytes

**Figure 6:**
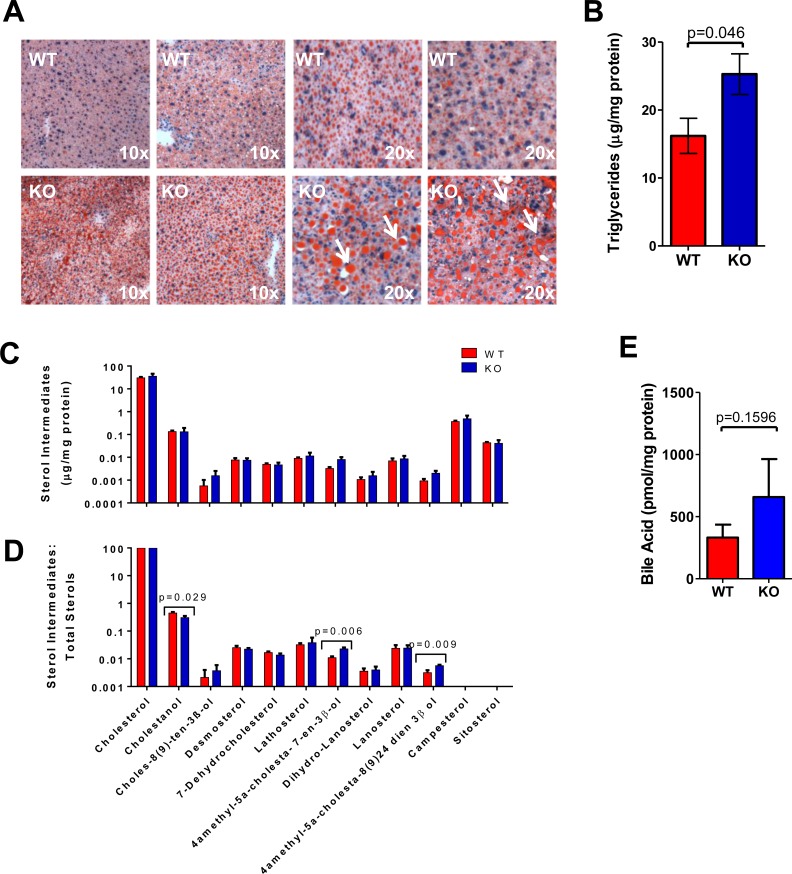
Hepatic repopulation enhances the defective handling of lipids in KO hepatocytes **A.** ORO-stained liver sections showing increased neutral lipid accumulation following transplant of KO hepatocytes. The last 2 sets of panels are higher power magnifications that demonstrate not only the overall increased lipid content in KO hepatocytes but the larger size of their lipid droplets and extracellular lipid deposits. See Figure S6C and S6D for quantification of lipid droplet numbers and sizes. **B.** Quantification of total triglyceride levels in mouse livers. Triglyceride assays were performed as described for Figure S5B. **C.** Liver sterol levels adjusted to protein content. Assays were performed as described for Figure S5E. **D.** Data from C expressed as a fraction of total sterol content as described in Figure S5F. **E.** No differences in the total bile acid of WT and KO transplanted liver, performed as described in Figure S5G. All assays were performed on at least 6 sets of tissue and analyzed by a *t*-test.

The proliferative stress imposed on donor hepatocytes might exaggerate previously identified abnormalities or reveal new ones. Consistent with this, ORO staining and triglyceride levels were again more pronounced in recipient livers transplanted with KO hepatocytes, with much of the stored lipid now residing extracellularly (Figure 6A and 6B). Interestingly, although the overall total number of lipid droplets/cell fell in transplanted KO hepatocytes, their average size increased, as did the overall cellular area occupied by lipid. These findings suggested that, following transplantation, the lipid droplets of KO hepatocytes increase in size by fusing with pre-existing droplets (compare Figure S6A and S6B to S6C and S6D).

As was the case pre-transplant (Figure S5E), sterol intermediates in recipients reconstituted with WT or KO hepatocytes were similar when normalized to protein content (Figure 6C) but differed when each analog, including the bile acid intermediate cholestanol, was expressed relative to the total sterol content (Figure 6D). Bile acid levels were also similar in the two groups (Figure 6E). These findings show that abnormalities in sterol biosynthetic pathways, initially identified in pre-transplant hepatocytes only by transcriptional profiling (Figure 3E) become more pronounced following transplantation.

### Transcriptional profiling of post-transplant hepatocytes

**Figure 7:**
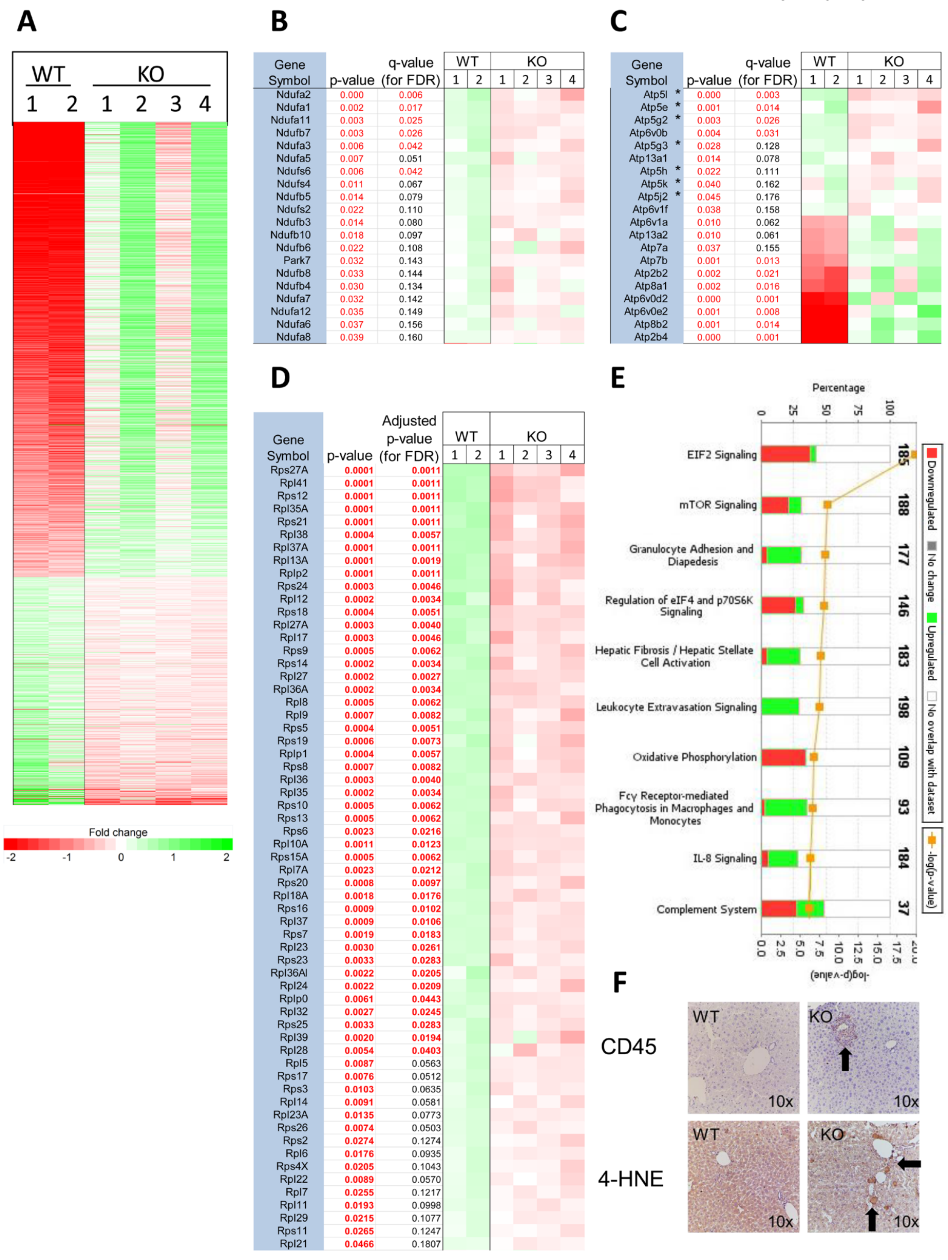
Transcriptional profiling of post-transplant hepatocytes **A.** RNAseq results for all 1784 differentially expressed transcripts with *q* values < 0.05 from isolated hepatocytes from 2 WT and 4 KO mice. **B.**-**D.** Gene ontology profiling for transcripts involved in common functions identified 20 encoding components of Complex I, 20 encoding various ATPases and 61 encoding ribosomal components. **E.** Ingenuity Pathway Analysis depicting the most de-regulated functionally related groups of transcripts shown in A. Green bars and red bars indicate transcripts that are up-regulated and down-regulated, respectively in cells isolated from animals transplanted with KO hepatocytes. Transcripts levels from these top 10 pathways are represented in Table S3.

Post-transplant transcriptional profiling is complicated given that isolated hepatocytes are a mix of donor and recipient cells (Figure 5B and 5C) that could potentially mask anything other than the most striking differences between the two populations. Any differences that are observed also cannot necessarily be attributed with certainty to the donor population. Nevertheless, with these caveats in mind, we repeated RNAseq on isolated liver cell populations from a small number of recipient mice ~18-19 weeks post-transplant. Unlike the limited number of transcript differences between pre-transplant hepatocytes (Figure 3A), we documented 1784 differences (*q* < 0.05) in the post-transplant setting (Figure 7A). Gene ontology analysis revealed that the 3 groups involving the greatest number of “common function” transcripts related to energy production, metabolism and ribosomal proteins. The first included 19 of the 44 known subunits of Complex I and Park7/DJ-1, which plays a role in mitochondrial fission and Complex I stabilization [35]. All these transcripts were down-regulated in the livers of mice repopulated with KO hepatocytes (Figure 7B). The second group contained 20 transcripts encoding subunits of various ATPases, 7 of which encoded components of Complex V (Figure 7C). These were down-regulated in the livers of mice repopulated with KO hepatocytes whereas 10 of the remaining 13 were up-regulated. The third group was comprised of transcripts encoding 61 members of both large and small ribosomal subunits, all of which were down-regulated (Figure 7D). This group contained all 6 small ribosomal subunit transcripts previously identified as being down-regulated in KO hepatocytes prior to transplant (Figure 3C).

6 of the 10 most highly altered pathways identified through Ingenuity Pathway Analysis, all of which were up-regulated in recipient livers transplanted with KO hepatocytes, pertained to some aspect of inflammation or healing, including the induction of fibrosis, leukocyte and IL-8 signaling, phagocytosis and modulation of the complement system, (Figure 7E and Table S3). Though the recipient mice were NOD-SCID and lacked T, B, and NK cells, they possess relatively normal levels of functional macrophages and neutrophils [34]. This immune response in recipients of KO hepatocytes strongly correlated with increased immuno-histochemical staining for the pan-leukocyte cell surface marker CD45 and for 4-hydroxynonenol, a by-product of lipid peroxidation (Figure 7F and S7). Thus, the livers of mice transplanted with KO hepatocytes show a pronounced expression of genes involved in acute inflammation and fibrosis as well as higher levels of inflammatory cells and oxidative damage.

The remaining 4 pathways were related by virtue of the central role of mTOR (mammalian target of rapamycin) in nutrient-sensitive control over protein translation and mitochondrial oxidative function, although mTOR also plays a central role immune modulation [36]. The most conservative interpretation of these results is that the livers of mice, otherwise equally repopulated with WT or KO hepatocytes, nevertheless vary considerably in their gene expression profiles, with the latter showing an up-regulation of transcripts involved in pro-inflammatory pathways and a down-regulation of transcripts regulating mitochondrial function and protein translation.

## DISCUSSION

Previous studies on Myc's role in hepatic function have produced conflicting or inconclusive results. For example, Baena *et al.* noted increased nuclear pyknosis and apoptosis in KO hepatocytes and a disorganized hepatic parenchyma with whereas Sanders *et al.* found no differences [11, 12]. Sanders *et al.* observed identical liver:body weight ratios in WT and KO mice whereas Qu *et al.* found them to be somewhat reduced in KO mice and Baena found them to be increased [11–13]. Only Sanders *et al.* reported liver weights following PH and found them to be similar in WT and KO mice [12]. Potential explanations for the discrepancies among these studies include variable efficiencies of *myc* gene knockout, excision of *myc* from cells other than hepatocytes and differences in animals' ages when various phenotypes were assessed. Longer term and/or subtler consequences of *myc* gene loss on proliferation could also not be assessed given that regeneration following PH is generally complete within 7-8 days [10].

Like Qu *et al*. [13], we found lower liver:body weight ratios in young KO mice (Figure S2A). Since WT and KO hepatocytes were of equivalent size (Figure S2C), the most likely explanation is that KO livers contained smaller numbers of normal-sized cells as previously described [8, 9]. The larger livers of older KO mice (Figure S1A) likely reflect their age-related lipid accumulation occurring as a result of their predisposition to this condition even at a young age (Figure S5 and S6A and S6B).

Increased VO_2_ by hemizygous (*myc+/−*) mice has been ascribed to a higher surface area:body mass ratio and/or to the increased activity and caloric intake of older animals [8]. These factors,, as well as the global nature of the *myc+/−* state, precluded a more definitive explanation for the higher VO_2_. In our KO mice, increased VO_2_ and VCO_2_ were not associated with differences in weight, caloric intake or activity compared to WT mice (Figure 1 and data not shown). Rather, they may reflect the greater reliance of KO hepatocytes on FAO as a source of energy (Figure S2H). The somewhat higher RER of KO mice maintained on a high fat diet also suggests these animals to be more reliant on glucose, possibly by peripheral tissues such as muscle, as is seen when fasted mice are re-fed a more carbohydrate-rich diet (Figure 1D).

The more pronounced metabolic abnormalities of KO mice maintained on a high fat diet is reminiscent of the metabolic changes observed in the hearts of obese and/or diabetic mice where reduced glucose uptake is offset by an imbalanced increase in fatty acid uptake and utilization, with the former exceeding the latter and the difference being stored as neutral lipid [37]. FAO is associated with more efficient production of TCA-cycle-derived NADH and FADH2, higher oxygen consumption and increased reactive oxygen species (ROS) production, which can cause mitochondrial uncoupling [38]. The greater activity of Complex V in KO liver mitochondria (Figure S3H and S3I) may therefore represent a successful compensatory mechanism to maintain normal ATP levels in the face of an excess reliance on FAO during a time of excessive ETC stress or compromise.

The abnormalities in fatty acid metabolism in KO mouse livers also bear similarity to those observed in *myc−/−* fibroblasts [20, 21]. That KO hepatocytes did not activate AMPK however suggests that, while energetically stressed, they maintain normal energy supplies, perhaps by optimizing their excessive reliance on FAO. This successful adaptation could be the result of less severe mitochondrial structural abnormalities than occur in *myc−/−* fibroblasts [21] (Tables S1 & S2 and Figure S3) and/or the higher Complex V activity (Figure S3H and S3I)[39]. It remains unclear why hepatocytes are so much less reliant on Myc than fibroblasts for maintaining mitochondrial integrity.

Unlike cell lines in which Myc over-expression affects hundreds-thousands of genes [40] KO hepatocytes had relatively few transcriptional changes (Figure 3A). This may reflect the relatively low expression level of Myc in the largely quiescent WT hepatocyte population and/or its otherwise normal regulation. Transcript differences included those encoding some of the same ribosomal proteins reported by others as being affected by Myc over-expression [12, 27]. However, the most striking differences involved pathways pertaining to sterol and bile acid biosynthesis. Cyp450 family members, which also play important roles in steroidogenesis and bile acid production, were also highly de-regulated [26, 41] (Figure 3E). Interestingly, 2 of the 7 most de-regulated pathways involved LXRs and FXRs, which are activated by oxysterol and bile acid metabolism, respectively [42]. LXRs and FXRs may also regulate fatty acid and glucose metabolism, both of which are also Myc-responsive [4, 28]. The lipid storage defects of KO livers thus appear to be the result of both increased fatty acid uptake [20, 24] and defective steroidogenesis. The ongoing accumulation of these in lipid droplets likely accounts for the age-related increase KO liver size (Figure S2A) [43].

Our repopulation experiments with WT and KO hepatocytes capitalized on the fact that they expand 50-100-fold following transplantation [16, 34] thus providing a more demanding and prolonged proliferative stress than afforded by PH and potentially uncovering previously unappreciated phenotypes [10, 34]. This model also allows a more accurate evaluation of intrinsic hepatocyte proliferative capacity given that it assesses re-population rather than regeneration which is more dependent on contributions from liver cells other than hepatocytes [10]. That the transplanted fraction remains constant over time and never fully replaces the recipient pool (Figure 5B and 5C) probably reflects the ability of donor cells to fully assume the disposal of toxic tyrosine catabolites, thus eliminating any further selective pressure. Collectively, these studies provide what we feel to be unequivocal proof that Myc is dispensable for hepatic repopulation, even under the most challenging circumstances.

Despite the equivalent repopulation potential of WT and KO hepatocytes (Figure 5), we observed marked differences in the post-transplant setting including an exacerbation of the pre-existing lipid abnormalities. The lipid accumulation by KO livers is reminiscent of non-alcoholic fatty liver disease that commonly evolves to a more severe form termed non-alcoholic steatohepatitis [32, 44]. This progression is associated with increased oxidative stress, de-regulation of cyp450 genes and extracellular lipid accumulation leading to long-term inflammation, fibrosis and hepatic failure [44, 45]. The prominence of inflammation- and fibrosis-related pathways in the post-transplant setting (Figure 7E) is consistent with such evolution and probably reflects the abundance of transcripts expressed by inflammatory cells that co-purify with KO hepatocytes (Figures 7F and S7). The widespread de-regulation of cyp450 genes in KO hepatocytes might further abet this pro-inflammatory environment by affecting arachidonate and eicosanoid metabolism [41].

Myc's dispensability for hepatocyte repopulation is consistent with some reports in other organ systems. For example, *myc* deletion during murine intestinal morphogenesis leads to an early but transient decline in the number of small intestinal crypts [46]. Long-term suppression of Myc in adult mice with a globally-expressed dominant-negative form of the protein is also associated with transient intestinal and hematopoietic phenotypes [7]. In contrast, *myc's* deletion from hematopoietic stem cells results in impaired hematopoietic renewal and a sustained reduction in both myeloid and lymphoid compartments [47]. The long-term survival and maintenance of normal tissues thus appears to be under distinct forms of Myc-directed control that in many cases are quite different than occur in tumors, which are commonly “addicted” to the oncoprotein [2, 7]. Clearly, it will be important in future work to define the nature of these differences.

## MATERIALS AND METHODS

### Animal studies

All animal work was conducted in conformity with the Public Health Service Policy on Humane Care and Use of Laboratory Animal Research (ILAR) Guide for Care and Use of Laboratory Animals. Experimental procedures were approved by the Institutional Animal Care and Use Committee at the University of Pittsburgh. C57BL6 c-Myc*^fl/fl^* (WT) mice were obtained as a gift from I. Moreno de Alboran [3, 11]. They were housed in a pathogen-free facility, maintained under standard conditions. Mice were fasted overnight before sacrifice unless otherwise noted. Myc KO mice were generated by crossing Alb-Cre recombinase-positive mice with WT (*myc^flox/flox^*) control mice, generating a hepatocyte specific KO mouse. All mice were genotyped using the PCR primers shown in Figure S1A-S1C and Table S4. FRG-NOD mice (Ark Pharm, Libertyville, IL) [34, 48] were bred and maintained on 8 mg/L NTBC (Yecuris, Inc., Tualatin, OR) in their drinking water. At the time of sacrifice, mice were euthanized, and their livers were weighed and divided into small pieces for further analysis.

### Hepatocyte isolation, transplantation into FRG-NOD mice and NTBC cycling

Hepatocytes were isolated as previously described using a two-step collagenase perfusion method [17]. Briefly, a catheter was inserted into the portal vein or inferior vena cava and 0.3-1.0 mg/ml collagenase II (Worthington Biochemical Corp, Lakewood, NJ) was circulated through the liver. Digested livers were placed in media (DMEM-F12 with 15 mM HEPES [Corning, Inc. Corning NY]) + 5% FBS, passed through a 70 um cell strainer and centrifuged at 55 x g for 3 min to remove non-parenchymal cells. Hepatocytes were washed 1-2 more times. Hepatocyte viability, determined by trypan blue staining, was typically > 80%. PCR primers listed in Table S4 allowed us to distinguish *fah^+/+^* donor and *fah^−/−^* recipient alleles. Hepatocytes were sized using a Vi-CELL Cell Viability Analyzer (Beckman Coulter, Indianapolis, IN) from at least 1500 hepatocytes from individual WT and KO mice. For transplantation, a total of 3 × 10^5^ donor (FAH*^+/+^*) hepatocytes were re-suspended in 100 μl of medium and injected intrasplenically into 6-8 week old FRG-NOD recipients. Transplanted mice were maintained on 8 mg/L NTBC for 4d following transplantation. NTBC was periodically withdrawn to promote proliferation of donor hepatocytes. Body weights were monitored and mice were re-started on NTBC when body weights dropped to < 80% of normal, minimizing morbidity and mortality. After several cycles of NTBC withdrawal, body weights stabilized at 100-120% of the initial weight, indicating high-level repopulation [16, 17].

### Histology and immunohistochemistry

H&E and oil red O (ORO) staining of paraffin-embedded sections were performed using standard histologic techniques. FAH immuno-staining was performed as previously reported [17]. Immuno-staining for Myc, CD45 and 4-HNE were performed under similar conditions (Table S5).

### Assays for pyruvate dehydrogenase, 3H-palmitate oxidation, acetyl CoA and ATP

All studies were performed as described previously [20, 25] except that they were modified for whole liver samples (approx. 100 mg each total weight). These assays were performed on at least 7 sets of WT and KO mouse livers.

### Immuno-blotting

Immuno-blotting was performed essentially as described previously using whole liver lysates [20, 25]. Antibodies used are listed in Table S5.

### Blue native gel electrophoresis (BNGE) of mitochondrial proteins and assays for ETC function

BNGE and enzymatic assays for ETC Complexes I, III, IV and V as well as supercomplexes comprised primarily of Complexes I, III and V were performed as previously described [21, 25] on 5 sets of isolated mitochondria from WT and KO mice.

### Targeted mass spectrometry assay for selected peptides and unbiased label free mass spectrometry assay

Mass spectrometry was performed on 5 sets of mitochondrial isolations from WT and KO mouse liver as previously described [25] with some differences as described in Supplemental Materials and Methods.

### Quantification of oxidative phosphorylation (Oxphos)

These studies were performed on an Oroboros Oxygraph 2k instrument (Oroboros Instruments, Innsbruck, Austria) and described further in the Supplemental Materials and Methods.

### Mitochondrial oxidoreductase assays

All oxidoreductase assays were performed using mitochondria isolated as described above for BNGE. Isocitrate dehydrogenase (IDH) activity was quantified in triplicate with 10 μg of protein as previously described using an IDH Activity Assay Kit [25]. Malic dehydrogenase (MDH) was measured on triplicate samples using Protocol SPOXAL01 (Sigma-Aldrich). Glycerol 3-phosphate dehydrogenase (G3PDH) was assayed in triplicate with a G3PDH Assay Kit (Abcam, Inc., Burlingame, CA) using 10 μg of total mitochondrial protein. All assays were performed on at least 8 sets of livers from WT and KO mice.

### RNAseq and analyses

RNA sequencing was implemented on 4 sets of hepatocytes isolated from WT and KO mice as previously described [49] with some minor differences as outlined in the Supplemental Materials and Methods.

### Hepatic triglyceride, sterol and bile acid quantification

Triglyceride content was determined on ~25 mg of flash-frozen tissue using the Infinity™ Triglycerides reagent (Thermo Scientific, USA) as previously described [50]. All assays were performed on at least 6 sets of tissue.

To determine serum and hepatic sterol levels, we used ion-ratio GC/MS on an Agilent 6390N/5973 GC/MS system on 7 sets of mice as previously described [51] with modifications to the GC/MS method to include ions specific for principal post-squalene cholesterol precursors. Bile acids were quantified on frozen total liver tissue using a Mouse Total Bile Acids Assay kit (Crystal Chem, Downer's Grove, IL) using the suppliers' protocol.

All statistical analysis was implemented using a simple Students' *t*-test unless otherwise noted.

## SUPPLEMENTARY MATERIALS FIGURES AND TABLES



## Abbreviations

FAH: Fumarylacetoacetate Hydrolase
NTBC: 2-(2-Nitro-4-Trifluoro-Methyl-Benzoyl)-1,3-Cyclo-Hexanedione
BNGE: Blue Native Gel Electrophoresis
IDH: Isocitrate Dehydrogenase
MDH: Malic Dehydrogenase
G3PDH: Glycerol 3-Phosphate Dehydrogenase
SDH: Succinate Dehydrogenase
PDH: Pyruvate Dehydrogenase
AMPK: AMP-Activated Protein Kinase
FAO: Fatty Acid β-Oxidation
ORO: Oil Red O (ORO)
FDR, q-value: False Discovery Rate
Cyp450: Cytochrome P450
FXR: Farnesoid X Receptor
LXR: Liver X Receptor
RER: Respiratory Exchange Ratio
VO2: Oxygen Consumption Rate
VCO2: Carbon Dioxide Production Rate
