# Supplementary material for: Abnormal lipid processing but normal long-term repopulation potential of myc−/− hepatocytes
Source: Oncotarget. 2016 Apr 20;7(21):30379–95. doi: 10.18632/oncotarget.8856 (PMC5058687; doi:10.18632/oncotarget.8856)
Supplement: Supplementary file 1 [file oncotarget-07-30379-s001.pdf]

Abnormal lipid processing but normal long-term repopulation potential of *myc-/-* hepatocytes

Supplemental Material and Methods

Metabolic cage studies. Mice were maintained on a diet comprised of 45 kcal% fat (D12451, Research Diets Inc., New Brunswick, NJ) for 22 weeks prior to performing metabolism studies (high fat diet). Mice (6-8 mice per group) were weighed every week and body composition was determined every four weeks in awake mice using EchoMRI (Echo Medical Systems, Houston, TX). Oxygen consumption (VO_2), respiratory exchange ratio (RER), and physical activity were determined using a Comprehensive Laboratory Animal Monitoring System (CLAMS, Columbus Instruments, Columbus, OH). Mice were fed-fasted-refed during the CLAMS monitoring. Re-feeding was with 5% glucose solution in addition to standard chow diet. The respiratory exchange ratio (RER) was defined as the ratio of VCO_2/VO_2. Caloric value (CV) was calculated from the formula: CV = 3.815 + (1.232 x RER) and heat production was calculated from the formula: Heat = CV x VO_2.

Assays for pyruvate dehydrogenase, ^{3}H-palmitate oxidation, acetyl CoA and ATP.

PDH activity was quantified using the PDH Enzyme Activity Microplate Assay Kit according to the directions provided by the supplier (MitoSciences, Eugene, OR). Liver pieces were homogenized in PBS and protein concentrations were determined using a BCA assay (Pierce, Inc., Rockford, IL). The homogenate was solubilized in the detergent provided in the kit and processed according the manufacturer's protocol. Typically, 100 µg of extract was assayed kinetically on a BMG LabTech FLUOstar Omega instrument for 30 minutes (Cary, NC). The kinetic rates were determined as change in OD over time.

For ^{3}H-palmitate oxidation, fresh tissue was finely minced and added to SET buffer (0.25 M sucrose, 10 mM Tris, 1 mM EDTA at pH 7.4) containing 1 mM carnitine plus with 0.5 µCi BSA-bound [9.10-^{3}H(N)]-palmitate (sp. act. = 32 mCi/mmol) (Perkin-Elmer, Waltham, MA). The mixture was incubated at 37C for

1 hour, then homogenized and subject to organic extraction as previously described [1]. Total protein content was determined using the BCA reagent.

To measure Acetyl CoA, a pulverized piece of flash frozen tissue was solubilized in ice cold lysis buffer (20 mM Tris, pH 7.5; 150 mM NaCl; 1 mM EDTA; 1 mM EGTA; 1% Triton X-100; 2.5 mM sodium pyrophosphate; 1 mM β-glycerolphosphate; 1 mM Na3VO4; 1 µg/ml Leupeptin and 1 mM PMSF). The assay was then carried out according to manufacturer's instructions using an Acetyl-Coenzyme A Assay Kit (Sigma-Aldrich cat. # MAK039).

ATP assays were performed by lysing tissues with 10% trichloroacetic acid to inactivate ATPases, and then diluting the samples 1:50 with PBS. 100 µl of this was mixed with 50 µl of ATPlite mammalian cell lysis solution in quadruplicate in 96 well plates. The remainder of the assay was carried out according to the manufacturer's instructions using the ATPlite^{TM} Luminescence Assay System (Perkin Elmer, Waltham, MA). Results were normalized to total protein levels, which were determined on separate sets of identical wells.

<u>Quantification of oxidative phosphorylation (Oxphos)</u>. Homogenates were prepared from fresh liver tissue as described in the protocol provided by the vendor (MiPNet17.02). Oxygen consumption was measured in MiR05 medium (0.5 mM EGTA, 3 mM $MgCl_2$, 60 mM K-lactobionate, 20 mM taurine, 10 mM KH_2PO_4, 20 mM HEPES, 110 mM Sucrose, and 1 g/L bovine serum albumin) at 37°C Substrates added throughout the experiment included those for Complex I (glutamate [10mM], malate [1mM], pyruvate [5mM]), complex II (succinate [10mM]), Complex V (ADP [4mM]) and cytochrome C (10 µM) to assess outer mitochondrial membrane integrity. O_2 consumption rates were normalized to total protein content. Ten sets of livers were analyzed on different days and later assessed statistically by a Paired t-test.

<u>In solution trypsin digestion for mass spectrometry</u>. For each digestion, 20 µg of mitochondria were re-suspended in 100 µl 50 mM NH4HCO3/0.02% ProteaseMAX™ Surfactant (Promega, Madison, WI). The mixture was then boiled for 10 min in the presence of 10 mM dithiothreitol and then alkylated by the addition of 45 mM iodoacetamide for 1 hr in the dark at room temperature). 0.5 µg of trypsin gold

(Promega, Madison, WI) was then added and digestion was performed overnight at 37°C. The resulting tryptic peptides were de-salted using PepClean C-18 Spin Columns (Pierce, Inc., Rockford, IL), vacuum-dried, and re-suspended in 40 µl of 0.1% formic acid.

Targeted mass spectrometry assays for selected peptides. Selective/multiple reaction monitoring (SRM/MRM)-based targeted mass spectrometry was performed on a TSQ Quantum Ultra instrument (Thermo Fisher Scientific) coupled to a Thermo Fisher Nanoflow Dionex Ultimate 3000 liquid chromatography system. Nano-LC separations, each performed in duplicate, used an analytical C18 PicoChip™ columns packed with 10.5 cm Reprosil C18 3µm 120Å chromatography media with a 75 µm ID column and a 15 µm tip (New Objective, Inc., Woburn, MA). Mobile phase A consisted of 0.1% formic acid in HPLC water and mobile phase B consisted of 0.1% formic acid in 100% acetonitrile. 2 µg of tryptic peptides were loaded onto the column and washed with mobile phase A for 4min at 6 µl/min. The peptides were then eluted into the analytical C18 column at a flow rate of 300 nl/min with a gradient comprised of 0-5% solvent B for 4.5 min, 5-40% solvent B for 31.5 min, 40-95% solvent B for 4 min and 95% solvent B for 8 min. The collision energies were calculated using a linear equation CE =0.034 x *m*/*z* + 3.314. The full width at half maximum was set to be 0.7 Da for Q1 and Q3. The instrument was operated using scheduled SRM mode with 1 sec cycling time and 5 min retention time window. The majority of peptides had base peak widths of ~20 sec base and ~12 data points were acquired per chromatogram peak. Skyline software [2] was used to facilitate targeted SRM assay method development and data analyses. The transitions of the targeted peptides were originally selected based on mouse tandem spectrum library downloaded from PeptideAtlas (http://www.peptideatlas.org/) and subsequently optimized on the TSQ. All selected peptides and their corresponding SRM assay parameters were listed in Supplemental Table S1. Peak areas of all transitions for the same peptide were summed and the total peak area was used as metric for relative quantitation. Student's t test was applied to log-transformed total peak area to determine the significance of the differences between groups.

Unbiased label free mass spectrometry assays. 2 µg of tryptic peptides per sample were analyzed with reverse-phased LC-MS/MS using a nanoflow LC (EASY-nLC II, Thermo Fisher) coupled online to

LTQ/Orbitrap Velos Elite hybrid mass spectrometer (Thermo-Fisher). Mobile phases contained 0.1% formic acid in HPLC grade water for solvent A and 0.1% formic acid in 100% acetonitrile for solvent B. Peptides were first loaded onto a C-18 trap column (Thermo Fisher) and desalted on line for 6 μl solvent A. Peptides were then eluted onto a capillary column (75 μm inner diameter x 360 μm outer diameter x 15 cm long (Polymicro Technologies, Phoenix, AZ) slurry-packed-in-house with 5 μm particle size, 125 Å pore size C-18 silica-bonded stationary phase (Phenomenex, Torrance, CA) and resolved using a 100 min gradient at the flow rate of 0.2 μl/min (3-33% B for 90min, 33-80% B for 2min, constant at 80% B for 6min, and then 80-0% B for 2min). Eluted peptides were analyzed via electrospray ionization to the mass spectrometer. Data was collected in positive ionization mode, with FT MS1 AGC targets = 100,000, maximum injection time = 200 ms, spray voltage = 2.5 kV, capillary temperature = 325°C. Acquisition consisted of cycle of a full scan FT mass spectrum at a resolution of 60,000 and top 20 MS/MS spectra recorded sequentially on the most abundant ions on the ion trap.

Relative label free quantification of identified peptides/proteins was obtained MaxQuant software (version 1.5.1.0). Acquired MS/MS spectra were searched using the MaxQuant build-in Andromeda search engine against a mouse proteome downloaded from UniProt (http://www.uniprot.org/) together with a sequence database for frequently encountered contaminants with the following modifications: trypsin as the proteolytic enzyme, static carbamidomethylation of cysteine (+57.0214 Da), variable oxidization of methionine (+15.9949 Da) and variable acetylation of proteins' N-termini (+42.0106 Da). Mass tolerance was set to 20 ppm for initial search and 4.5 ppm for the main search for precursor ions, and 0.5 Da for fragment ions. Minimum peptide length was set to 7 amino acids and the false discovery rate (FDR) based on a target-decoy approach was set to 1%. The match between runs options was enabled with 2 min match time window and 20 min alignment window. The intensity values from MaxQuant output for identified peptides were used for relative quantitation across samples. Only peptides with non-zero intensity values in all samples were included for statistical test. Student's t test was applied to log-transformed intensities to identify peptides with significant difference between WT and KO. The linear step-up (LSU) [3] was used to compute FDR-adjusted p-values (q values) to account for multiple comparisons. Mitochondrial localization was confirmed by the David

(http://david.abcc.ncifcrf.gov/) Bioinformatics Database and/or mouse MitoCarta Inventory (http://www.broadinstitute.org/pubs/MitoCarta/mouse.mitocarta.html). All selected peptides and their corresponding SRM assay parameters were listed in Supplemental Table S2.

RNAseq and analyses. Total RNAs were extracted from 10^7 isolated WT or KO hepatocytes using Qiagen RNeasy columns (Valencia, CA). Each RNA sample was assessed for quality and quantity using a Qubit 2.0 fluorometer and Agilent Bioanalyzer TapeStation 2200. The Sample preparation was replicated using Illumina TruSeq Stranded mRNA kit (San Diego, CA). mRNA was used to generate poly-A+ libraries using oligo-dT beads following 2 rounds of poly-A selection. Following this, RNA was fragmented to an average length of 260 nt by magnesium-catalyzed hydrolysis at 94^0C and then converted into cDNA by random priming. cDNA 3' ends were adenylated, followed by adaptor ligation and a 9-cycle PCR to enrich DNA fragments. cDNA libraries were then validated using KAPA Biosystems (Wilmington, MA) primer premix kit with Illumina-compatible DNA primers on the Qubit 2.0 fluorometer and Tapestation 2200 systems described above. Libraries were diluted in 0.1% Tween 20 to concentrations of 2 nM for stabilization. The cDNA libraries were then pooled to final concentrations of 2.5 pM. Cluster generation and 75 bp paired-end single-indexed sequencing were performed with an Illumina NextSeq 500 sequencer. Sequence analysis was performed using mRNA-Seq for Differential Expression in Eukaryotes (Maverix Biomics, San Mateo, CA). Raw sequencing reads were quality checked for potential sequencing issues and contaminants using FastQC (Babraham Bioinformatics, Inc, Cambridge, UK.). Adapter sequences, primers, Ns, and reads with quality score below 28 were trimmed using fastq-mcf of ea-utils and PRINSEQ (http://prinseq.sourceforge. net/manual.html). Reads with a remaining length of less than 20 bp after trimming were discarded. Paired end reads were mapped to the mouse genome (m10) using TopHat (http://ccb.jhu.edu/software/tophat/index.shtml) in a strand specific manner. Read coverage on forward and reverse strands for genome browser visualization was computed using SAMtools, BEDtools, and UCSC Genome Browser utilities. Pairwise differential expression was quantified using Cuffdiff and DESeq [4, 5]. Cufflinks was used to determine FPKM levels for each gene from the TopHat alignment and was used as input for Cuffdiff. Significant differentially expressed genes were determined by adjusted P-value with a threshold of 0.05. DESeq was utilized

for raw read counts, which were calculated by HTSeq based on the TopHat alignment (http://www-huber.embl.de/users /anders/HTSeq/doc/count.html). Read counts were then normalized across all samples and significant differentially expressed genes were determined by adjusted P-value with a threshold of 0.05 [3].

Supplemental References

1. Edmunds LR, Sharma L, Kang A, Lu J, Vockley J, Basu S, Uppala R, Goetzman ES, Beck ME, Scott D and Prochownik EV. c-Myc programs fatty acid metabolism and dictates acetyl-CoA abundance and fate. The Journal of biological chemistry. 2014; 289:25382-25392.
2. MacLean B, Tomazela DM, Shulman N, Chambers M, Finney GL, Frewen B, Kern R, Tabb DL, Liebler DC and MacCoss MJ. Skyline: an open source document editor for creating and analyzing targeted proteomics experiments. Bioinformatics. 2010; 26:966-968.
3. Benjamini Y and Hochberg Y. Controlling the false discovery rate: a practical and powerful approach to multiple testing. Journal of the Royal Statistical Society Series B (Methodological). 1995:289-300.
4. Trapnell C, Hendrickson DG, Sauvageau M, Goff L, Rinn JL and Pachter L. Differential analysis of gene regulation at transcript resolution with RNA-seq. Nature biotechnology. 2013; 31:46-53.
5. Trapnell C, Williams BA, Pertea G, Mortazavi A, Kwan G, van Baren MJ, Salzberg SL, Wold BJ and Pachter L. Transcript assembly and quantification by RNA-Seq reveals unannotated transcripts and isoform switching during cell differentiation. Nature biotechnology. 2010; 28:511-515.

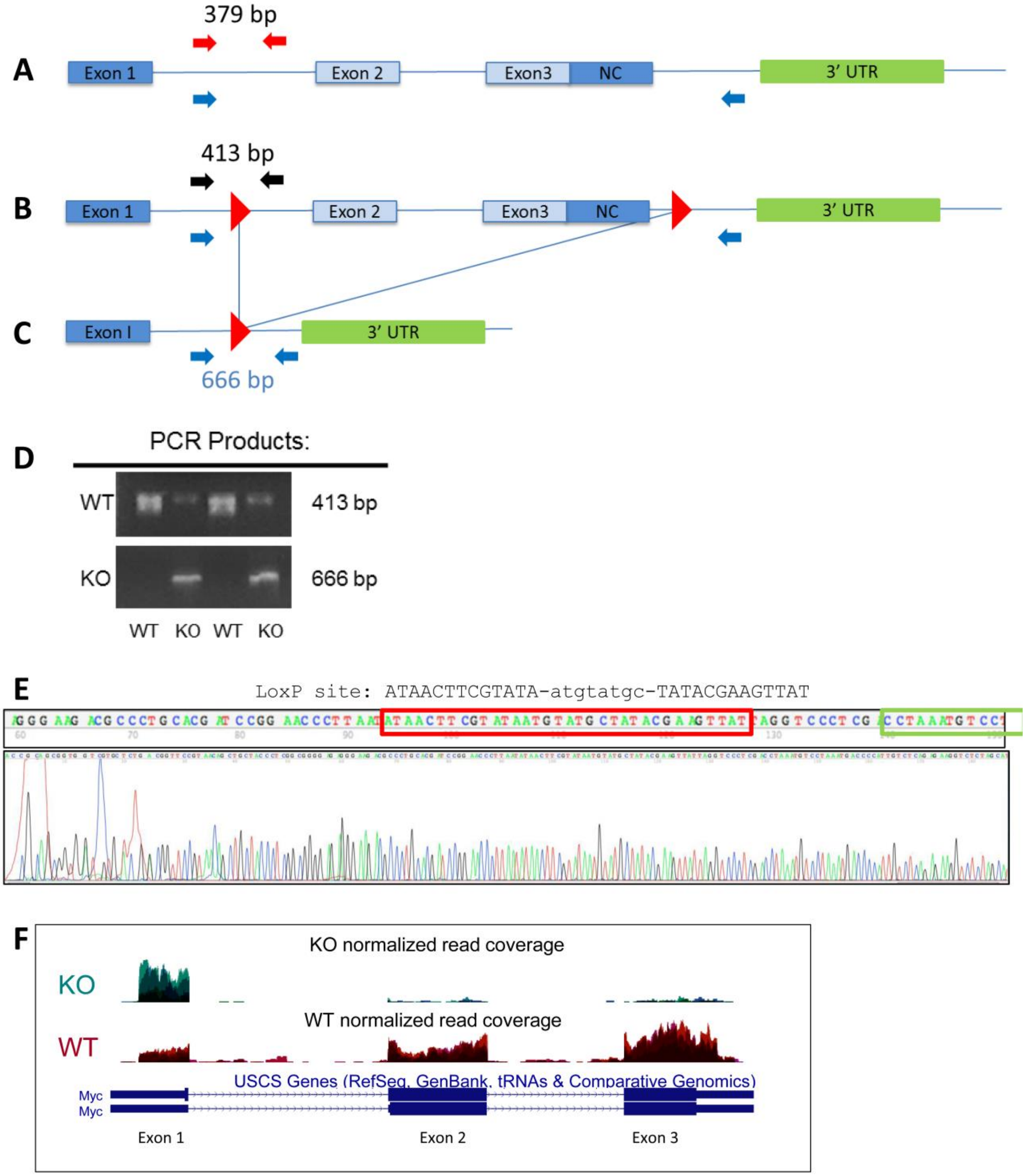

Gene ID	Gene	Description	WT abundance	KO abundance	Fold Change	p-value
17869	Myc	myelocytomatosis oncogene	200.23	34.07	-2.3	2.9×10^{-9}

Supplemental Figure S1. Deletion of *myc* coding exons 2 and 3 from KO hepatocytes. (A). The *myc* locus in “unfloxed” WT (*myc*$^{wt/wt}$*)* animals. Red arrows indicate the same set of primers as black arrows in B but with a smaller product. (B). The *myc* locus in WT (*myc*$^{fl/fl}$*)* animals. LoxP sites are indicated by red triangles. PCR primers used to amplify the WT allele are indicated with black arrows with the size of the expected PCR product (in bps) indicated. (C). The *myc* locus following Cre-recombinase-mediated recombination. PCR primers used to amplify KO (*myc-/-*) alleles are indicated by blue arrows with the size of the PCR product indicated. (D). *myc* alleles were amplified from WT and KO hepatocyte DNAs of 3 month old mice and resolved by agarose gel electrophoresis. Typical genotyping results are shown for two representative animals of each group. (E). DNA sequence of the relevant region of the KO PCR product, confirming the deletion and showing that the LoxP region (boxed in red) is flanked by intron 1 and intron 3 sequences. Note that the PCR primers depicted in A can also be used to distinguish this allele from a normal *myc* allele without LoxP sites. (F). KO hepatocytes show greatly reduced expression of *myc* coding exons 2 and 3. RNAseq reads across each of the 3 *myc* exons were compiled for both WT and KO hepatocytes. Each set of reads represents the sum of sequences generated from hepatocyte RNAs of four animals from each group.

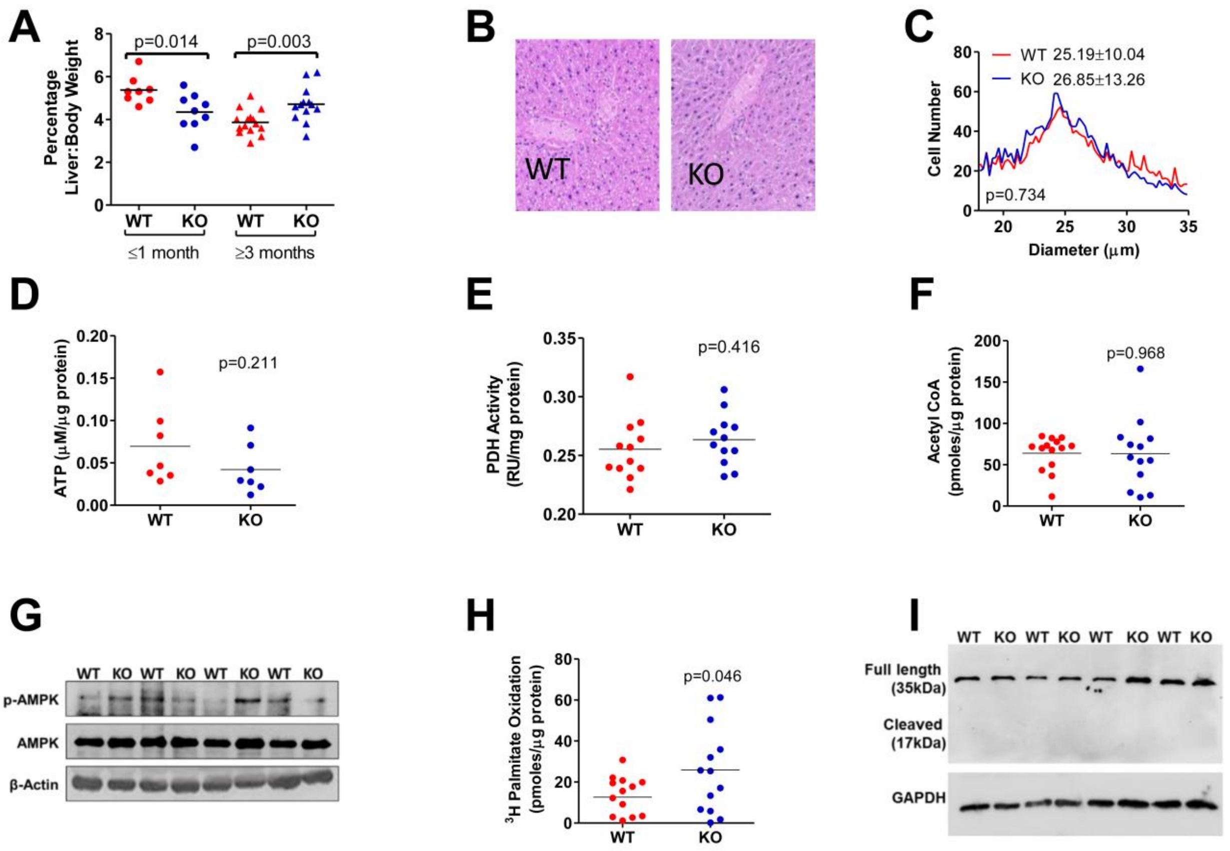

Supplemental Figure S2. Characterization of WT and KO livers and hepatocytes. (A). Liver:body mass ratios. Weights were determined on groups of young and old mice (<1 month and >3 months old, respectively; n≥8). (B). Typical histologic appearance of H&E-stained sections of WT and KO livers from young mice. No obvious differences were noted in examination of >10 livers from each group. (C). Hepatocyte sizing from ~2 month old WT and KO mice. At least 1500 hepatocytes from individual WT and KO mice were examined. Similar results were obtained from two additional sets of animals (not shown). (D-F). ATP content, PDH activity and acetyl CoA levels, respectively, were quantified on liver lysates (n≥7) as previously described [1]. (G) AMPK Immunoblotting. Total liver lysates from individual WT or KO mice were immuno-blotted for total AMPK, pAMPK(Thr_{172}) and β–actin as a loading control. (H) ^{3}H-palmitate β-oxidation. Liver slices (n=13) were incubated with ^{3}H-palmitate as previously described [1] and the amount of tritium incorporated into water-soluble products

was quantified. (I). Caspase 3 immunblots. Total liver lysates from WT and KO mice were blotted against full length and cleaved Caspase 3 with GAPDH as a loading control.

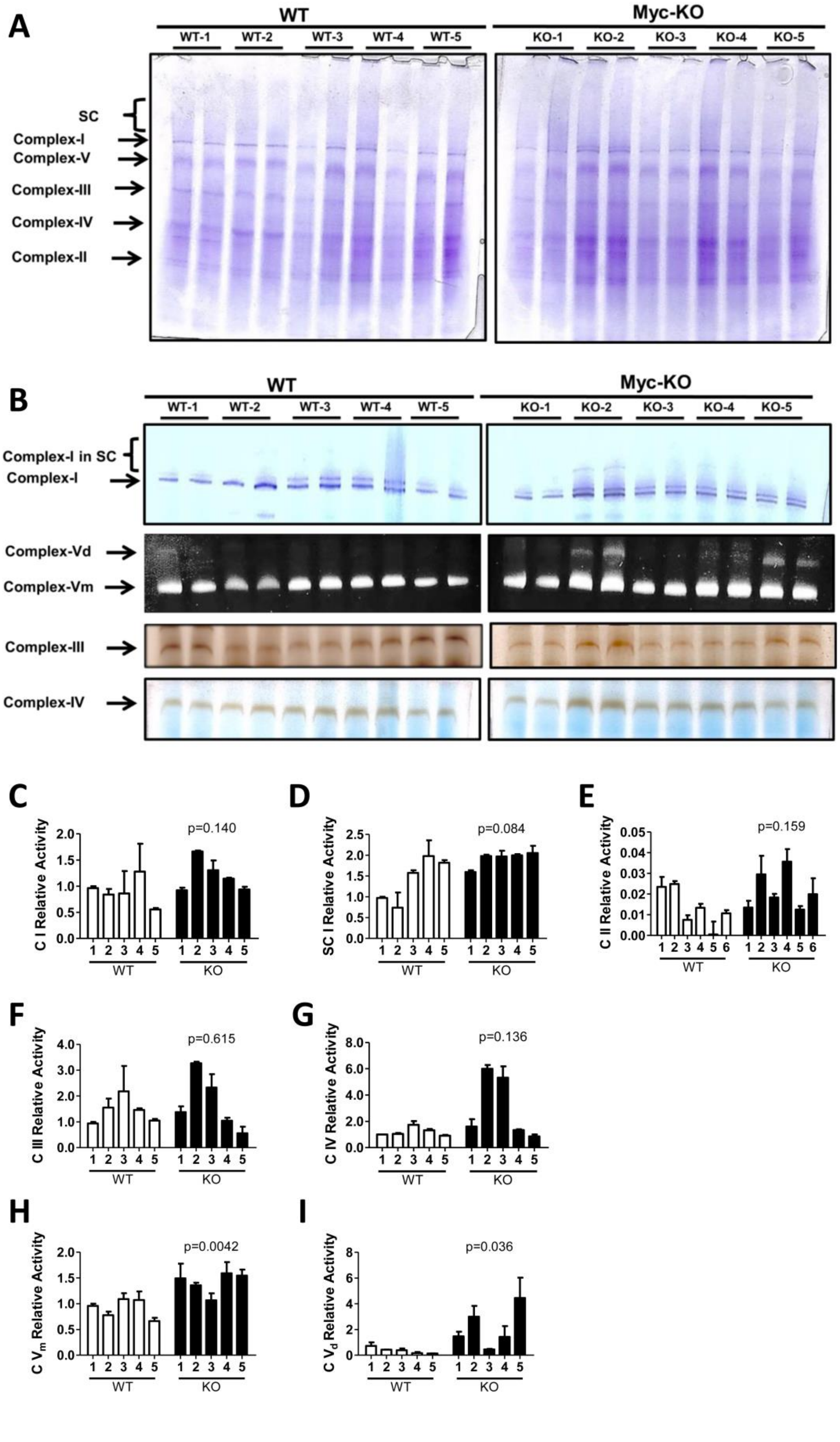

A
WT
Myc-KO
WT-1
WT-2
WT-3
WT-4
WT-5
KO-1
KO-2
KO-3
KO-4
KO-5
SC
Complex-I
Complex-V
Complex-III
Complex-IV
Complex-II
B
WT
Myc-KO
WT-1
WT-2
WT-3
WT-4
WT-5
KO-1
KO-2
KO-3
KO-4
KO-5
Complex-I in SC
Complex-I
Complex-Vd
Complex-Vm
Complex-III
Complex-IV
C
C I Relative Activity
p=0.140
WT
KO
D
SC I Relative Activity
p=0.084
WT
KO
E
C II Relative Activity
p=0.159
WT
KO
F
C III Relative Activity
p=0.615
WT
KO
G
C IV Relative Activity
p=0.136
WT
KO
H
C Vm Relative Activity
p=0.0042
WT
KO
I
C Vd Relative Activity
p=0.036
WT
KO

Supplemental Figure S3. Comparison of ETCs in WT and KO livers. (A). Representative BNGE of duplicate mitochondrial samples, each prepared independently from 5 WT and 5 KO mouse livers. Mitochondria were purified as previously described [2, 3]. Arrows indicate each of the complexes as well as higher order supercomplexes (SCs) comprised primarily of different stoichiometries of complexes I, III and V [4-7]. (B). Examples of typical *in situ* enzymatic assays for each of the indicated SCs. Relevant regions of gels such as those shown in A were excised and assayed *in situ* under the conditions described in Supplemental Materials and Methods. Complex II could not be measured *in situ* and was instead measured separately on isolated mitochondrial lysates as described previously [1, 8]. *In situ* enzyme activities for each of the indicated complexes are depicted here graphically after adjusting for differences in the protein content of each complex based on densitometric scanning of BNGE profiles [3]. (C). Complex I activity. (D). Complex I activity in SCs. (E) Complex II activity. (F) Complex III activity. (G) Complex IV activity. (H) Complex V_m activity. (I). Complex V_d activity. Each bar depicts the results of triplicate assays performed on mitochondria isolated from independent liver samples of the same animal. Standard errors and p-values were calculated based on the mean of all results.

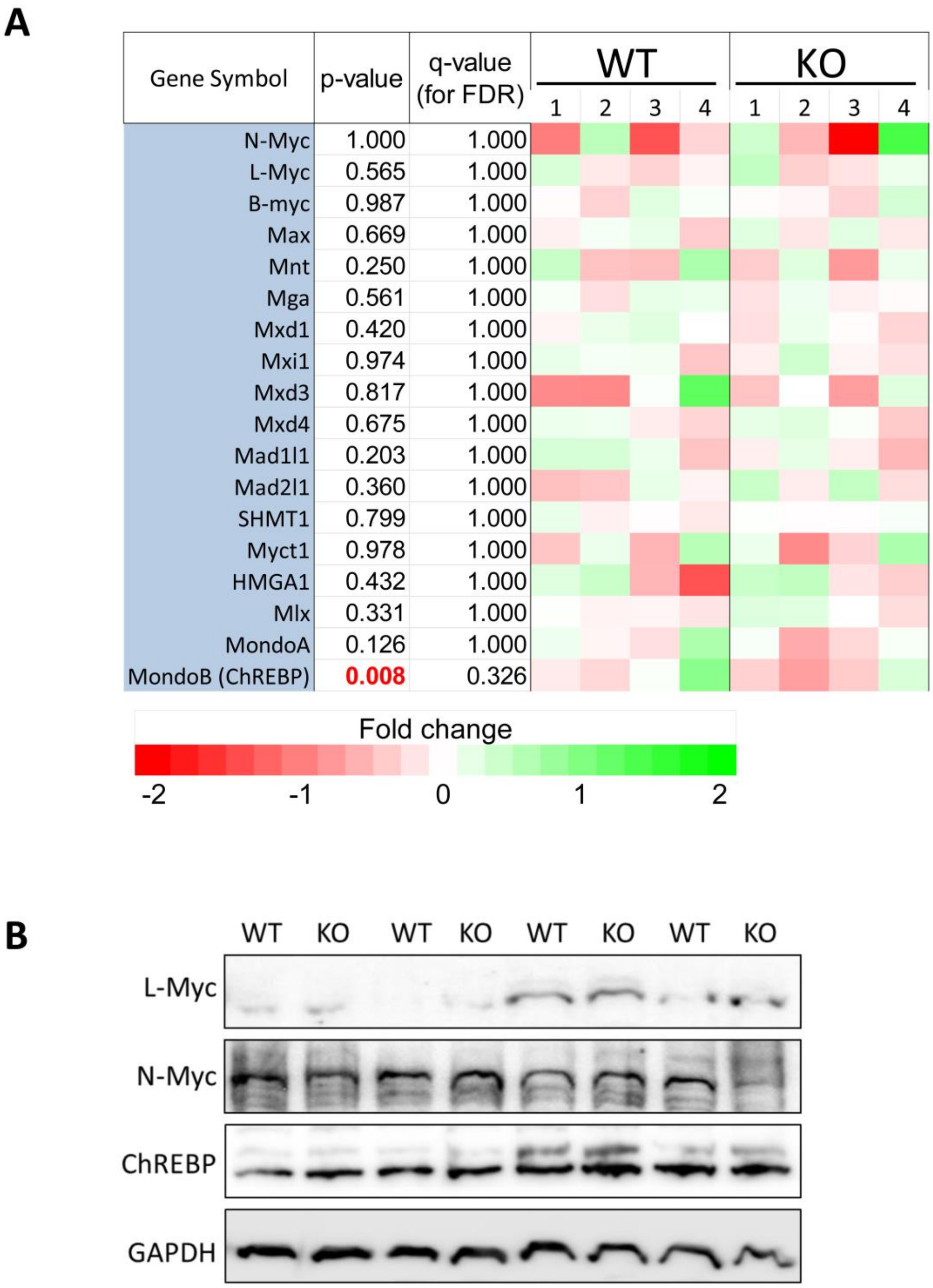

Supplemental Figure S4. Lack of differential expression of most transcripts encoding Myc homologs, negative regulators of Myc function and proteins that can restore some or all Myc phenotypes in *myc-/-* fibroblasts. (A). The results shown here were extracted from RNAseq data discussed in Figure 3. Note the modest but significant down-regulation of

Mlxip1/MondoB/ChREBP expression in KO livers. (B). Immuno-blots for N-Myc, L-Myc, and ChREBP proteins with GAPDH as a loading control.

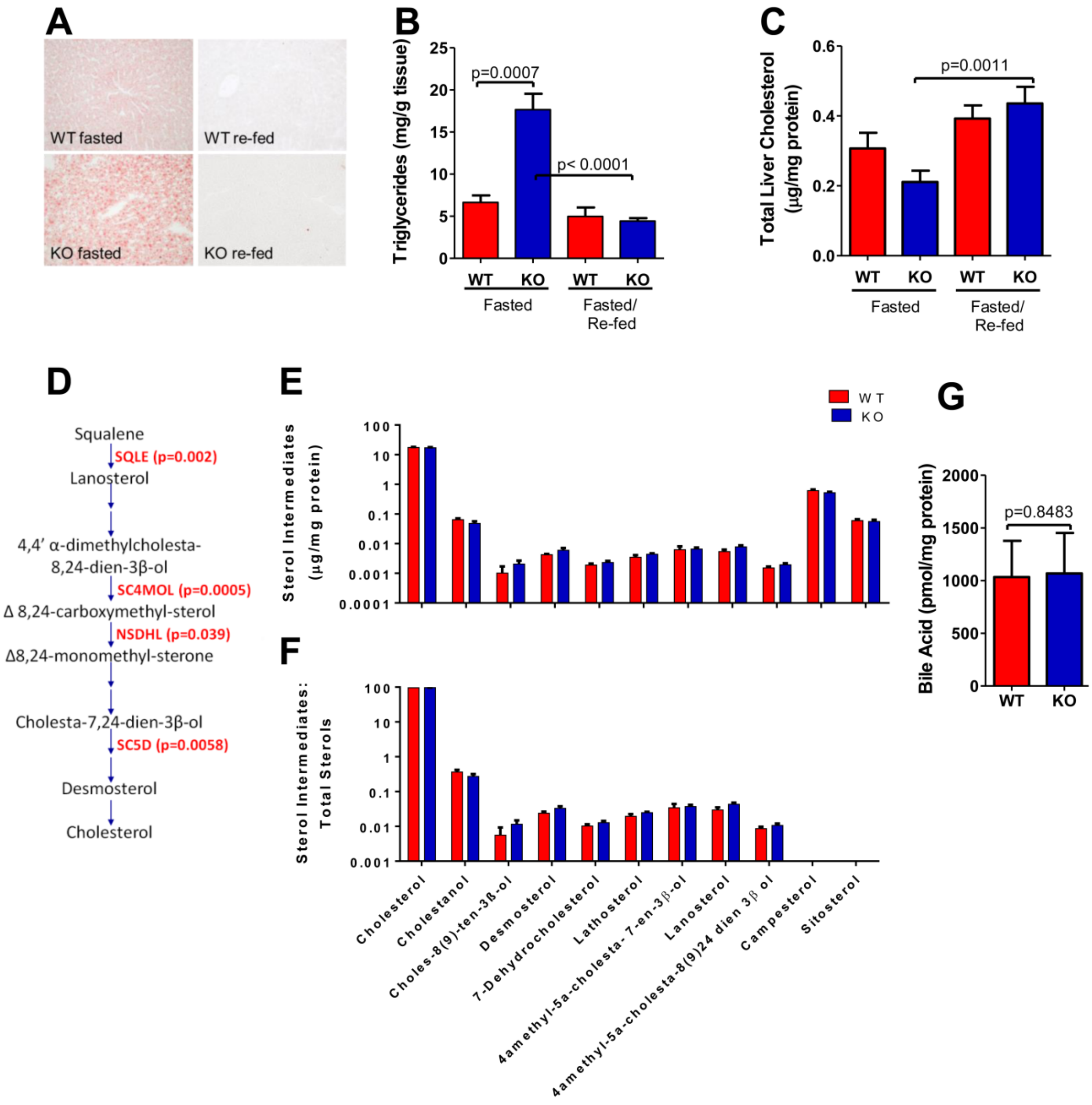

Supplemental Figure S5. Triglyceride, sterol and bile acid levels in WT and KO livers. (A). Representative ORO-stained liver sections after fasting or fasting + re-feeding. (B). Quantification of total liver triglycerides in at least 6 representative animals from each of the indicated groups. (C). Quantification of total cholesterol levels in at least 6 animals from each of the indicated groups. (D). Minimal biosynthetic pathway from squalene to cholesterol showing a select sub-set of relevant sterol intermediates and enzymes (red, p-values indicated in parenthesis) whose transcripts were up-regulated in KO hepatocytes as indicated by RNAseq. (E). Liver sterol levels adjusted to protein content (n=7) [9, 10]. (F). The data from (E) expressed as a fraction of total sterol content. (G). Total bile acids measured in livers of starved mice (n=6).

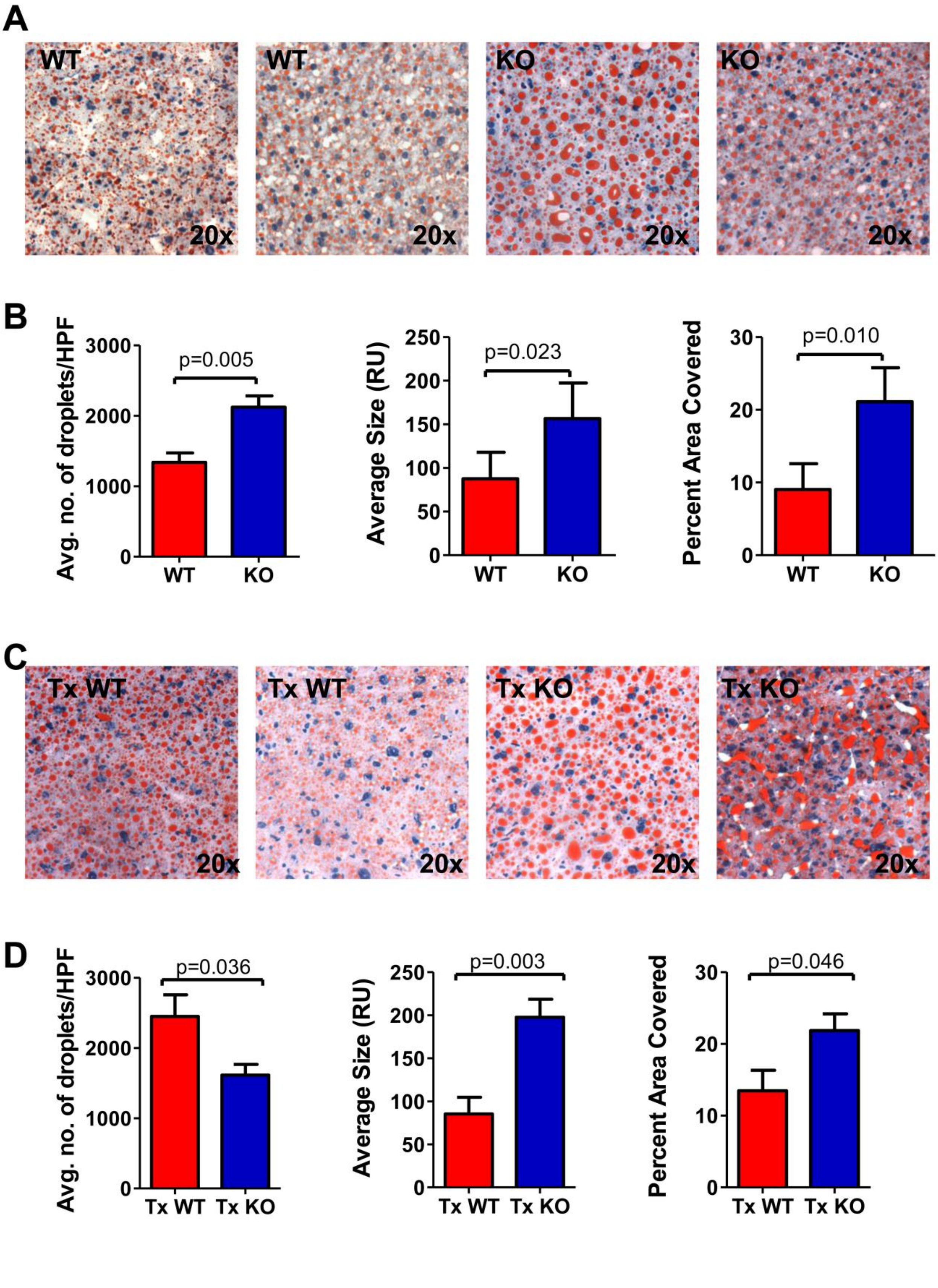
A
WT
WT
KO
KO
20x
B
Avg. no. of droplets/HPF
3000
2000
1000
0
p=0.005
WT
KO
Average Size (RU)
250
200
150
100
50
0
p=0.023
WT
KO
Percent Area Covered
30
20
10
0
p=0.010
WT
KO
C
Tx WT
Tx WT
Tx KO
Tx KO
20x
D
Avg. no. of droplets/HPF
p=0.036
Tx WT
Tx KO
Average Size (RU)
p=0.003
Tx WT
Tx KO
Percent Area Covered
p=0.046
Tx WT
Tx KO

Supplemental Figure S6. Lipid droplets in KO hepatocytes are more numerous and larger. (A). Shown are representative images similar to those depicted in Supplemental Figure S5A on which analyses were performed. (B). Lipid droplet number, size and total average percent area occupied within hepatocytes was determined using ImageJ software (Analyze Particles function). Analyses were performed on a total of 5 mice from each group with a total of 5 independent images being taken from each histologic section. HPF=high power field. RU=relative units. (C). Representative images of lipid droplets in hepatocytes of recipient livers following transplantation with WT or KO hepatocytes. (D). Quantification was performed as described in B.

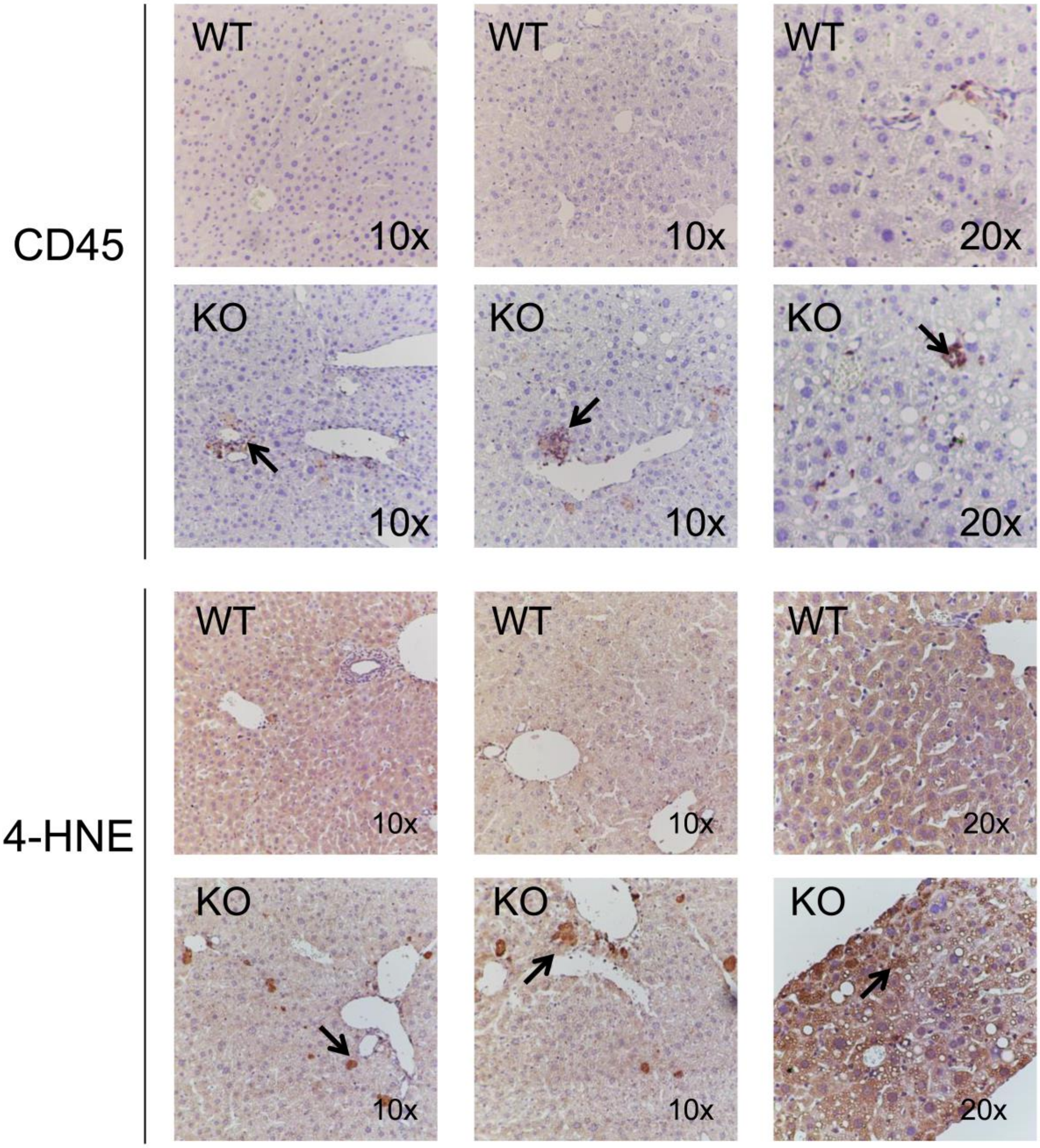

Supplemental Figure S7. Post-transplant immunohistochemical staining of recipient livers for CD45 and 4-hydroxynonenal (4-HNE). Each image is typical of the remaining section and is taken from individual mice (n=5). Arrows indicate representative areas of positivity.

Supplemental Table S1. LC-MS/MS-based quantification of ETC component proteins and other targeted proteins from WT and KO livers. Total peak areas from 271 peptides identified through PeptideAtlas are compared from mitochondria isolated from the livers of 5 WT and 5 KO mice. Both a Students t-test and a Wilcoxon signed-rank test were used to compare the relative abundance of the peptides corresponding to 139 proteins.

Supplemental Table S2. LC-MS/MS-based quantification of non-ETC mitochondrial proteins from WT and KO livers. High resolution LC-MS/MS data was acquired for the samples indicated in Supplemental Table S1. The resulting MS data were analyzed using the differential mass spectrometry tools (InfoClinika, Seattle WA) that are included in the CHORUS data analysis environment (www.chorusproject.org). Total peak areas from 2439 peptides were collected from 5 individual liver mitochondrial preparations from each group and matched to 377 mitochondrial proteins using the PeptideAtlas. All results were compared using both a Students t-test and Wilcoxon signed-rank test.

Supplemental Table S3. Transcripts identified by Ingenuity Pathway Analysis from the top 10 deregulated pathways in transplanted livers. Differential gene expression profiling of genes identified as described for Figure 3C, performed on isolated hepatocytes from 2 WT mice and 4 KO mice.

Table S4: Description of the primers used throughout the manuscript.

Table S5: Description of the antibodies used throughout the manuscript.

References

1. Edmunds LR, Sharma L, Kang A, Lu J, Vockley J, Basu S, Uppala R, Goetzman ES, Beck ME, Scott D and Prochownik EV. c-Myc programs fatty acid metabolism and dictates acetyl-CoA abundance and fate. The Journal of biological chemistry. 2014; 289:25382-25392.
2. Wang Y, Mohsen AW, Mihalik SJ, Goetzman ES and Vockley J. Evidence for physical association of mitochondrial fatty acid oxidation and oxidative phosphorylation complexes. The Journal of biological chemistry. 2010; 285:29834-29841.
3. Graves JA, Wang Y, Sims-Lucas S, Cherok E, Rothermund K, Branca MF, Elster J, Beer-Stolz D, Van Houten B, Vockley J and Prochownik EV. Mitochondrial structure, function and dynamics are temporally controlled by c-Myc. PloS one. 2012; 7:e37699.
4. Acin-Perez R and Enriquez JA. The function of the respiratory supercomplexes: the plasticity model. Biochimica et biophysica acta. 2014; 1837:444-450.
5. Barrientos A and Ugalde C. I function, therefore I am: overcoming skepticism about mitochondrial supercomplexes. Cell metabolism. 2013; 18:147-149.
6. Genova ML and Lenaz G. Functional role of mitochondrial respiratory supercomplexes. Biochimica et biophysica acta. 2014; 1837:427-443.
7. Vartak R, Porras CA and Bai Y. Respiratory supercomplexes: structure, function and assembly. Protein & cell. 2013; 4:582-590.
8. Munujos P, Coll-Canti J, Gonzalez-Sastre F and Gella FJ. Assay of succinate dehydrogenase activity by a colorimetric-continuous method using iodonitrotetrazolium chloride as electron acceptor. Analytical biochemistry. 1993; 212:506-509.
9. Kelley RI. Diagnosis of Smith-Lemli-Opitz syndrome by gas chromatography/mass spectrometry of 7-dehydrocholesterol in plasma, amniotic fluid and cultured skin fibroblasts. Clinica chimica acta; international journal of clinical chemistry. 1995; 236:45-58.
10. Kelley RI, Wilcox WG, Smith M, Kratz LE, Moser A and Rimoin DS. Abnormal sterol metabolism in patients with Conradi-Hunermann-Happle syndrome and sporadic lethal chondrodysplasia punctata. American journal of medical genetics. 1999; 83:213-219.
